# Reproductive outcomes in women and men using complementary and alternative medicine treatment and not receiving artificial reproductive technology: a systematic review

**DOI:** 10.1007/s00404-020-05836-4

**Published:** 2020-10-20

**Authors:** Hannah M. Yogasundram, Andrew J. O. Hui, Clifford Y. S. Sia, Anthea C. Chui, William J. Waldock, Siobhan Quenby, Elizabeth Brown, Clare Oliver-Williams

**Affiliations:** 1 School of Clinical Medicine, University of Cambridge, Cambridge, UK; 2 Warwick Medical School, University of Warwick, Coventry, UK; 3 University Hospitals Coventry and Warwickshire, Coventry, UK; 4 University College London, London, UK; 5 Homerton College, University of Cambridge, Hills Road, Cambridge, UK; 6 Department of Public Health and Primary Care, University of Cambridge, 2 Worts’ Causeway, Cambridge, CB1 8RN UK

**Keywords:** Pregnancy, Conception, Live births, Miscarriage, Complementary medicine, Alternative medicine

## Abstract

**Purpose:**

Infertility is a global problem, but only a minority of couples access assisted reproductive technologies due to financial and sociocultural barriers. Complementary and alternative medicine are seen as another option. We aimed to determine the impact of complementary and alternative medicine on conception, miscarriage and live birth rates in couples not receiving assisted reproductive technology treatments.

**Methods:**

The electronic databases EMBASE, PubMed, Web of Science and the Allied and Complementary Medicine Database were systematically searched before March 24th 2020. Reference lists of eligible studies were searched for relevant studies. Eligible studies included trials and observational studies that assessed a complementary or alternative medicine and conception, miscarriage or live births in men or women not undergoing fertility treatment. Data were extracted by two independent reviewers using a pre-designed data collection form. The study protocol was published in the PROSPERO database (CRD42018086980).

**Results:**

Twenty randomized controlled trials were identified, including 2748 individuals. Most studies did not demonstrate any effect of a complementary or alternative medicine on pregnancy, live birth or miscarriage rates. Limited evidence was found for a positive effect of herbal therapies taken by women on conception rates. There was substantial diversity in quality across the studies.

**Conclusion:**

There is limited evidence of the effectiveness of complementary and alternative medicine on improving the chances of conception and live births, or increasing miscarriage risk. Owing to the generally sub-optimal quality and heterogeneous nature of the evidence, rigorous studies are needed to determine the impact of complementary and alternative medicine on fertility.

**Electronic supplementary material:**

The online version of this article (10.1007/s00404-020-05836-4) contains supplementary material, which is available to authorized users.

## Introduction

Infertility is a significant problem which affects 7–16% of couples seeking to have children [1, 2]. Assisted reproductive technology (ART) treatment is regularly used to help couples experiencing infertility to have a child. However, inequity in access to ART treatment have been well reported, [3–5] and there is evidence for a declining trend in the use of ART treatment in the US [6].

Given the disproportionate access to ART treatment as well as its potentially high cost, alternatives are often sought by couples. Use of complementary and alternative medicines (CAM) to treat infertility is common. This may be because people perceive CAM to be of lower cost, safer or more effective [7]. A 2010 prevalence study from eight American community and academic infertility practices found that 29% of couples had used a CAM for infertility, with 22% trying acupuncture [8].

There is a broad range of CAM that purport to have a positive effect on fertility including traditional practices such as acupuncture: Chinese medicine remedies; use of naturopaths, homeopaths and chiropractors; and cognitive and mind–body therapies, such as mindfulness. Although the potential impact of these treatments on infertility has been assessed within the context of ART treatment [9], this has limited interpretation for couples who are not receiving ART treatment.

Considering the increasingly wide-ranging use of CAM by couples trying to conceive and the inequity in access to ART treatment, a comprehensive review on the efficacy of CAM treatments in couples not receiving ART treatment is essential. Therefore, we present a systematic review of all available intervention and observational studies to review the impact of a range of CAM on conception, miscarriage or live births rates in couples not receiving ART treatment.

## Methods

### Information sources and search strategy

This review was conducted using a predefined protocol, in accordance with PRISMA guidelines (eTable 1) and is registered on PROSPERO [10] (CRD42018086980). Four electronic databases (EMBASE, PubMed, Web of Science and the Allied and Complementary Medicine Database) were searched until 24th March 2020 without time and language restriction. The computer-based searches combined terms related to (1) the interventions, such as alternative or complimentary natural therapies (including *herbal therapies, traditional Chinese medicine, ayurveda, naturopathy, chiropractic, osteopathy, massage, yoga, relaxation therapy, homeopathy, aromatherapy and therapeutic touch*) (2) pregnancy and related outcomes (e.g., *conception, miscarriage, birth*) and (3) study design (e.g. *RCT, longitudinal study*) in humans (eTable 2). CAM were identified through previous CAM publications [11–13] to aid comparison and popular fertility websites which provide advice to couples (https://www.fertilityiq.com; https://www.babycentre.co.uk; https://www.oviahealth.com). Two independent reviewers (EB, WW, AC, HY, CS, AH) screened the titles and abstracts of all studies initially identified according to the selection criteria, and any disagreement was resolved through consensus or consultation with a third independent reviewer (CO). Full texts were retrieved from studies that satisfied all selection criteria. Reference lists of studies and reviews identified on the topic were searched to identify additional publications.

### Selection criteria

Studies were eligible if they: (1) were longitudinal studies, randomized controlled trials (RCTs) or non-randomized trials; (2) assessed effects of any CAM (list in eTable 3) in men or women seeking to conceive; (3) collected relevant endpoints, including conception, miscarriage or live births rates; and (4) control and treatments groups only differed on the administration of a CAM. Study populations included women or men experiencing sub-fertility or infertility due to a diagnosed medical condition (e.g. polycystic ovary syndrome (PCOS), diminished ovarian reserve) or due to an unexplained or unspecified condition. The participants could not be receiving in vitro fertilization (IVF) or another ART treatment at the time of the trial. No restrictions on length of follow-up or language were applied.

### Data extraction

The exposures or interventions eligible for inclusion in the current review were summarized using the following broad groupings: (i) biologically based therapies such as herbal extracts and dietary supplements not conventionally recommended for conception or pregnancy (i.e. folic acid would not be eligible); (ii) mind–body and behavioral therapies (e.g. mindfulness); (iii) body manipulation and energy therapies (e.g. acupuncture); and (iii) traditional (alternative) medical systems (e.g. homeopathy). Two authors independently extracted data (EB, WW, AC, HY, CS, AH) and a consensus was reached in case of any inconsistency with involvement of a third author (CO). A predesigned electronic data abstraction form was used to extract relevant information. The form included the following: author, year, study design, country, study years, number of participants, number lost to follow-up, recruitment process and inclusion/exclusion criteria, CAM, category of CAM (Biological/Energy/Body manipulation/Traditional medical systems), control group intervention, outcome(s), the number receiving the CAM, follow-up duration, average age of participants, pregnancies in treatment/control groups (*N* or %), miscarriages in treatment/control groups (*N* or %), live births in treatment/control groups (*N* or %), risk estimate for the difference between the therapy and control group, adverse events in the CAM group and an open text field for additional notes. In case of multiple publications on a study, the most up-to-date or comprehensive information was extracted.

### Statistical analysis

For studies which did not statistically compare the rates of the outcome by exposure group the 2-sided chi-squared test was conducted using the raw data provided in the manuscript. Analyses were conducted Stata version 12.0 (Stata Corp, College Station, Texas, USA). All P values from 2-sided tests were considered statistically significant at *p* < 0.05.

### Assessing the risk of bias

Two reviewers independently rated the quality of studies using the Cochrane Collaboration’s tool (HMY, CO) [14]. This tool evaluated seven domains for their risk of bias (high, low, or unclear) as follows: random sequence generation, allocation concealment, blinding of participants and personnel, blinding of outcome assessment, incomplete outcome data, selective reporting and other biases. We provided an overall assessment of the risk of bias in each study as follows: if a study had no domains considered at high risk and at least four domains deemed low risk (at least one of them being random sequence generation or allocation concealment), the study was deemed low risk; if a study had one domain at high risk it was deemed to be a high risk study; otherwise it was deemed to be of unclear risk. Any disagreements were discussed until a consensus was reached.

To assess the overall quality of evidence, two authors applied the Grading of Recommendations Assessment, Development and Evaluation (GRADE) approach. As individual studies were assessed using the Cochrane Collaboration’s tool, assessment of the GRADE approach was only applied where two or more studies were identified for an exposure–outcome combination. We downgraded the evidence from high quality by one level for each serious study limitation that was identified. Study limitations were the following: risk of bias, indirectness of evidence, serious inconsistency, imprecision of effect estimates or potential publication bias. Two or more studies were identified for the following exposure and outcome combinations and were, therefore, included in the GRADE assessment: antioxidants for men and conception; herbal therapies for women and conception, acupuncture for women and conception; and homeopathy for women and live births.

Meta-analyses were not conducted in part because the majority of studies did not assess the same exposure and outcome. The few studies that did evaluate the same exposure–outcome combination had important methodological heterogeneity, including study quality, population studied and study design, which was reflected in statistical heterogeneity in a preliminary meta-analysis.

## Results

**Fig. 1:**
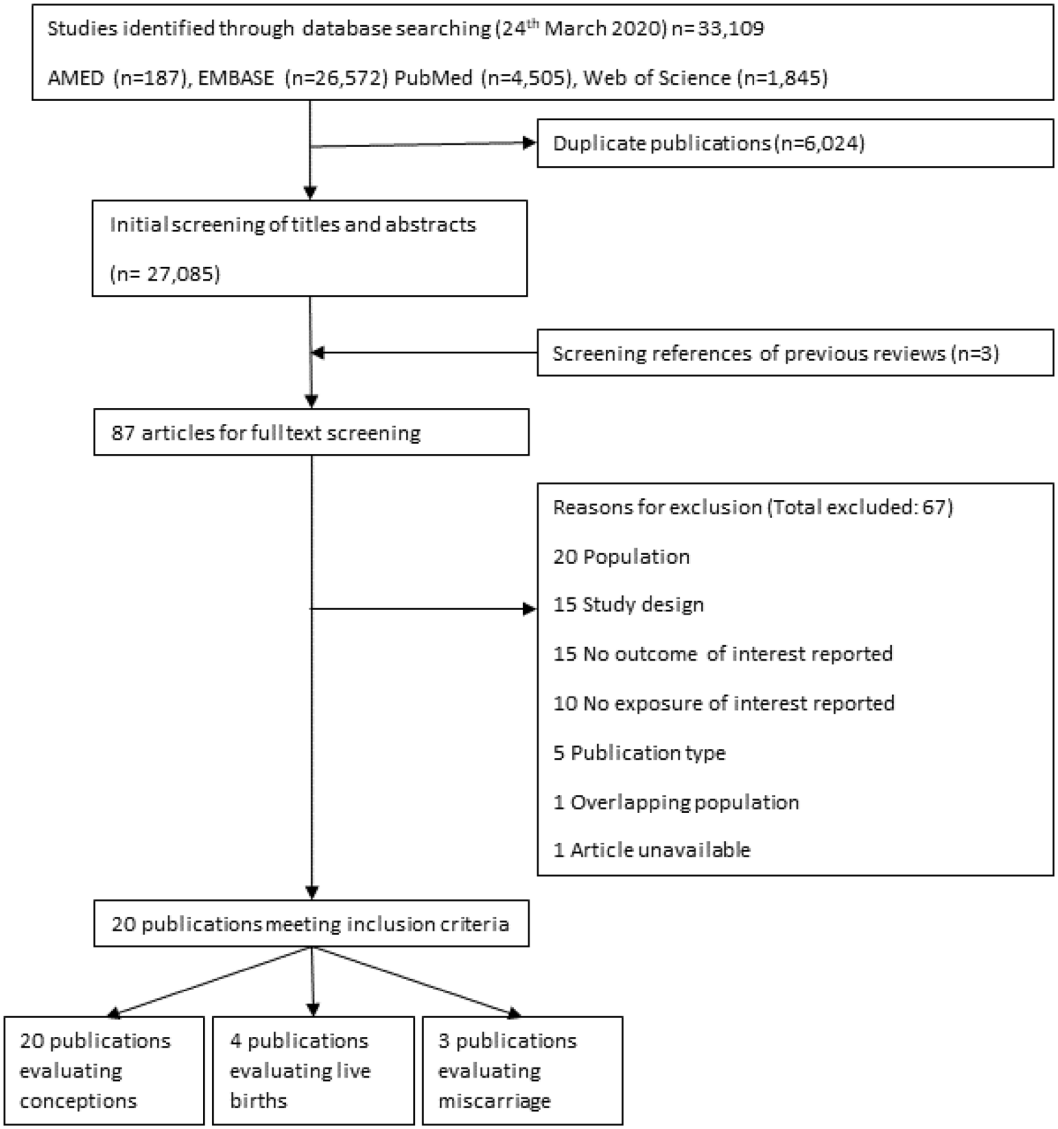
Flow diagram of identification and selection of studies included in the systematic review

**Fig. 2:**
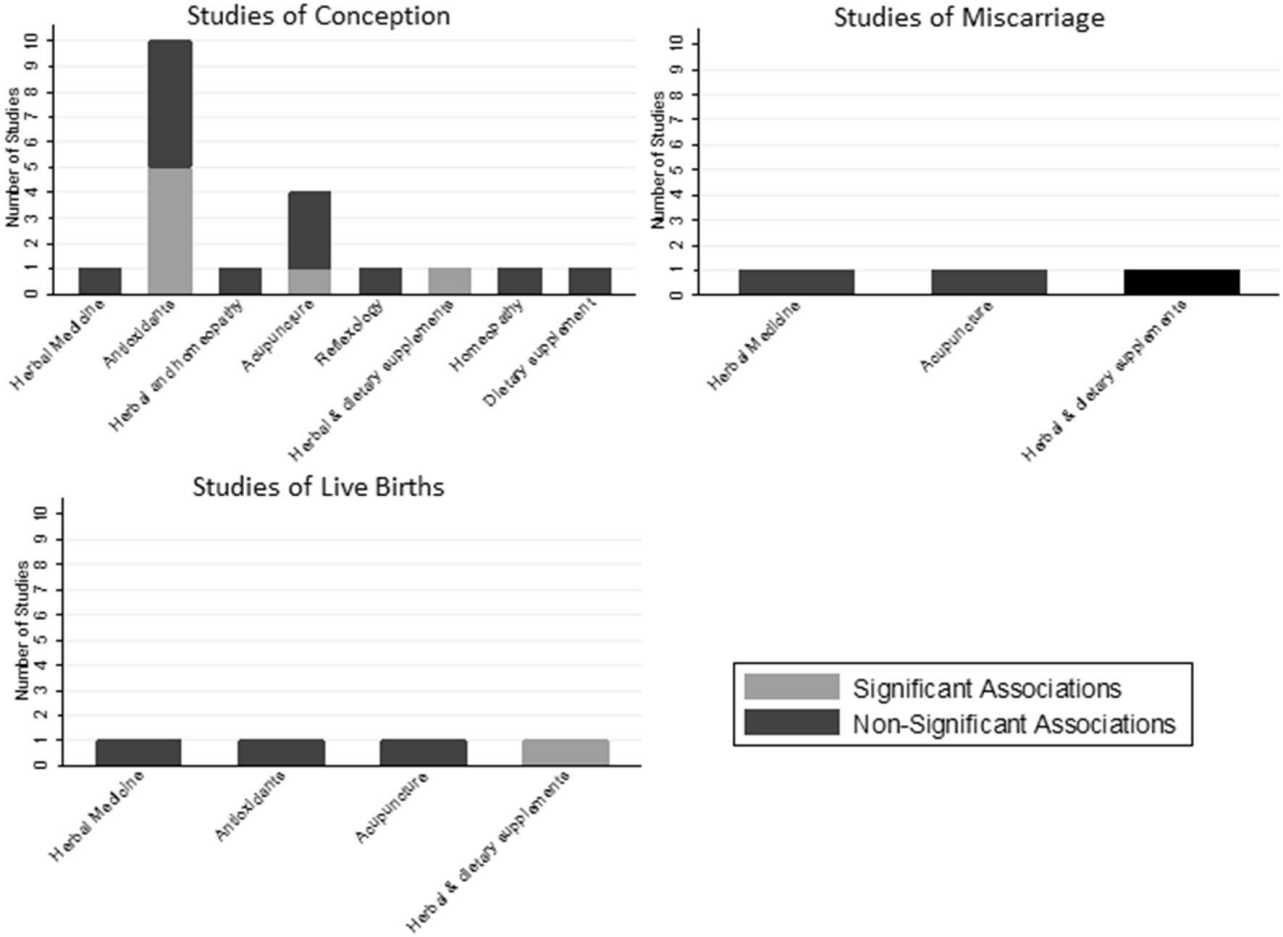
Summary of the included studies results

We identified 27,085 publications in our search strategy after removal of duplicates. After assessment, 20 original articles were identified that evaluated conception, miscarriage or live births after the use a CAM (Fig. 1) [15–34]. Table 1, Fig. 2 and eTable 4 summarize the studies included in the review. The results of these studies are presented by endpoint in order of the direction of evidence. The quality of evidence from these studies is summarized in Table 2. The interventions and results of the studies evaluating conception, live births and miscarriages are summarized in eTables 5–7, respectively. The included studies evaluated a biological CAM (*n* = 13), energy therapies (*n* = 4), body manipulations (*n* = 1), traditional CAM (*n* = 1) or a combination (*n* = 1).

**Table 1:** Description of studies included in the review

Author, year	Complementary or alternative medicine	Outcomes	Study years	Country	Study duration and follow-up	Follow up duration (months)	Number of participants recruited (*n*)	Mean age (years)	Description of population
Arentz [27]	Biological—Herbal medicine	Conception, miscarriages, live births	2012-onwards	Australia	< 3 months treatment	0	122	29	Overweight women with PCOS, 18–44 years
Balercia [17]	Biological—Antioxidants	Conception	Not given	Italy	1 month run-in, 6 months of therapy or placebo, 3 months follow-up	3	60	30	Sexually active men with idiopathic and varicocele-associated oligoasthenospermia, aged 20–40 years
Balercia [15]	Biological—Antioxidants	Conception	Not given	Italy	1 month run-in, 6 months of therapy or placebo, 3 months follow-up	3	60	32	Sexually active men with idiopathic asthenozoospermia, aged 20–40 years
Bergmann [21]	Biological/Traditional—Herbal and homeopathic treatment	Conception, live birth	1993–1996	Germany	3 months or 3 cycles of treatment, 6 months follow-up	6	78	30	Women with oligomenorrhoea or amenorrhoea, aged 18–40 years
Busetto [31]	Biological—Antioxidants	Conception	12/2014–6/2015	Italy	6 months of supplement or placebo. follow up 1 cycle post treatment and 1 visit 6 months later	6	104	33	Men (18–50 years) with oligo/astheno/teratozoozpermia, infertile for > 1 year and no other fertility related diseases. Must have a fertile female partner (< 40 years), not currently seeking ART treatment within the next 90 days
Cavallini [28]	Biological—Antioxidants	Conception	1999–2002	Italy	6 months of treatment, and 3 months further follow-up	3	380	Not given	Sexually active men unable to conceive after 1 year, aged 27–44 years
Cochrane [24]	Energy—Acupuncture	Conception	2009–2011	Australia	3 months of treatment, 12 months follow-up	12	56	Not given	Women unable to conceive for at least 12 months, aged 18–44 years
Gopinath [16]	Biological—Antioxidants	Conception	Not given	India	180 days of treatment, up to 6 months follow-up	6	138	31	Sexually active men who have been subfertile for 1 + years, aged 21–50 years
Holt [26]	Body manipulation—Reflexology	Conception	2001–2003	UK	10 weeks of treatment, 2 months of follow-up	2	49	Not given	Women with oligomenorrhea: < 6 menstrual periods in the last 12 months, or with low luteal phase progesterone, aged 18–38 years
Lenzi [29]	Biological—Antioxidants	Conception	1998–2000	Italy	8 months treatment (2 months washout, 2 months treatment, 2 months washout, 2 months treatment) 2 months follow-up	2	100	Not given	Sexually active men with unexplained infertility for 2 + years, aged 20–40 years
Lenzi [30]	Biological—Antioxidants	Conception	Not given	Italy	2 months washout, 6 months of treatment, 2 months follow up	2	60	Not given	Sexually active men with unexplained infertility for 2 + years, aged 20–40 years
Lim [33]	Energy—Acupuncture	Conception	2008–2009	China and Australia	1 month run in, 3 months of treatment, 3 months follow-up	3	146	26	Women with PCOS, complete amenorrhea and with Kidney Yang deficiency pattern of Chinese medicine syndrome, aged 20–33
Pastore [22]	Energy—Acupuncture	Conception	2006–2009	USA	2 months of treatment, 3 months follow-up	3	96	27	Women with PCOS, aged 18–43 years
Razavi [32]	Biological—Antioxidants	Conception	October 2014–December 2014	Iran	3 months without oral contraeptives, then 8 weeks treatment or placebo	0	64	25	Women (18–40 years) with a PCOS diagnosis (defined by oligomenorrhoea + high androgens); > 1 menses in past 6 months ; < 8 menses in last year; no hormonal contraceptives, metformin or fertility medication for 5 months duration of study
Scott [19]	Biological—Antioxidants	Conception	Not given	Scotland	3 months treatment, 2 weeks after end produce sperm sample	0.5	69	33	Men with reduced sperm motility who had been attending a subfertility clinic
Sigman [18]	Biological—Antioxidants	Conception	Not given	USA	24 weeks, no follow-up	0	26	36	Men with infertility for 6 + months, aged 18–65 years
Westphal [34]	Biological—Herbal and dietary supplement	Conception, miscarriages, live births	Not given	USA	3 months of treatment	0	30	35	Women unable to conceive after 6–36 months, aged 24–46 years
Witmann [25]	Traditional—homeopathic	Conception	Not given	Germany	3 months treatment	0	84	30	Women wanting to conceive, tubal patency, positive post coital test, normal spermiogram, no hormonal treatment in last 3 months, no anatomical abnormalities, > 18 years
Wu [23]	Energy—acupuncture	Conception, miscarriages, live births	2012–2014	China	Up to 16 weeks of treatment, 10 months of follow-up	10	1000	28	Women with PCOS, aged 20–40 years
Závaczki [20]	Biological–—dietary supplement	Conception	Not given	Hungary	90 days of treatment, no follow-up	0	26	29	Sexually active men with unexplained infertility for 1 + years, and a diagnosis of pathospermia

**Table 2:** Quality of evidence evaluating complementary and alternative medicine use and reproductive outcomes

Effect	Quality of evidence
Antioxidants for men do not affect rates of conception^a^	Very low (D)
Herbal therapies for women improve rates of conception^b^	Very low (D)
Acupuncture for women does not affect rates of conception^c^	Low (C)
Homeopathy for women has no statistically significant effect on live birth rates^d^	Low (C)

GRADE rating was applied when two or more studies assessed the same exposure and outcome. GRADE rating—C: low, the true effect might be markedly different from the estimated effect; D: very low, the true effect is probably markedly different from the estimated effect

^a^Based on ten studies

^b^Based on three studies;

^c^Based on four studies;

^d^Based on two studies

### Conception

Twenty studies investigated conception after treatment with a CAM (eTable 5**)**. All studies were RCT. Eleven studies were carried out in Europe, three in the USA, two in Australia, one each in China, Iran, India and one in China and Australia together. Women and men were treated in ten studies each. Thirteen studies assessed biological based therapies, four studied energy therapies, one each studied body manipulation and traditional methods and one assessed a combination of body manipulation practices and traditional CAM. Of the biological therapies, there were ten antioxidants, one herbal supplement, one dietary supplement and one combined herbal and dietary supplement.

Seven studies found evidence for a positive effect of CAM treatment on conception and 12 did not show a difference.

### Studies reporting no statistically significant effect

Six studies found no statistically significant effect of antioxidants in sexually active men experiencing infertility. Balercia et al. [15] studied the effect of co-enzyme Q10 (200 mg/day) taken for 6 months with 3 months’ follow-up in a placebo-controlled, double-blind RCT of 60 sexually active men with idiopathic infertility. In the treatment group, six partners became pregnant compared to three in the control group (20.0% vs 13.0% *p* = 0.301).

In a factorial, placebo-controlled, double-blind RCT, Balercia et al. [17] assessed the effect of l-carnitine (3 g/day), l-acetyl-carnitine (3 g/day), or combined l-carnitine (2 g/day) and l-acetyl-carnitine (1 g/day) over a 6-month period with 3 months’ follow-up on 60 men with idiopathic infertility. Two partners became pregnant in each of the single therapy groups, compared to three in the control group (13.3% and 13.3% vs 20.0%, *p* = 0.624). Five partners became pregnant in the combined therapy group (33.3% vs 20.0%, *p* = 0.409).

Gopinath et al. [16] evaluated the effect of antioxidants on 138 men with subfertility in a three-arm, double-blind, placebo-controlled RCT. Men received either two tablets of antioxidants a day (50 mg co-enzyme Q10, 500 mg l-carnitine, 2.5 g lycopene, 12.5 mg zinc), one tablet of antioxidants and one placebo tablet, or two placebo tablets for 180 days, with up to 6 months of follow-up. There were six conceptions for men receiving two tablets of antioxidants, seven for men receiving one tablet of antioxidant and two in the control group, (13.0% vs 5.6%, *p* = 0.257) and (16.3% vs 5.6%, *p* = 0.135), respectively.

Sigman et al. [18] evaluated the effect of 24 weeks of l-carnitine (2 g/day) and l-acetyl-carnitine (1 g/day) on men in a randomized, double-blind placebo-controlled trial with no follow-up. Of the 26 men recruited, one man from the control group impregnated his partner (11.1% vs 0%, *p* = 0.237).

A three-arm, double-blind RCT by Scott et al. [19] compared the effects of (1) l-selenomethionine (100 μg/day), (2) l-selenomethionine with vitamins and (3) a placebo taken daily for 3 months on 69 subfertile men. There was 2 weeks’ follow-up. Five men in total achieved paternity in the two treatment groups compared to none in the placebo group (10.9% vs 0.0%, *p* = 0.145).

Závaczki et al. [20] recruited 26 sexually active men to a placebo-controlled RCT of Magnesium-orotate (3 g/day) for 90 days with no follow-up. One partner in the treatment group became spontaneously pregnant (10.0% vs 0.0%, *p* = 0.305).

Bergmann et al. [21] assessed the effects of 3 months’ treatment with a homeopathic preparation of the herbal therapy Agnus castus, in a placebo-controlled, double-blind RCT that recruited 78 women with infrequent or absent menstruation. There was 6 months’ follow-up after treatment. Two spontaneous pregnancy were found in the treatment group and one in the control group (6.7% vs 3.3%, *p* = 0.554).

Three studies found no statistically significant effect of acupuncture on pregnancy rates. In a double-blind, sham-controlled trial, Pastore et al. [22] randomized 96 women with PCOS to receive either 8 weeks of true or sham acupuncture with 3 months of follow-up. Of those who were eligible to become pregnant (*n* = 45), four in the acupuncture group and three in the control group became pregnant (20.0% vs 12.0%, *p* = 0.680).

Wu et al. [23] conducted a placebo-controlled, blinded factorial trial evaluating the effect of 16 weeks of acupuncture and clomiphene on women with PCOS who were trying to conceive. They were followed-up for 10 months after treatment. Of the 1000 women recruited, there was no difference in the number of pregnancies when comparing women who received acupuncture and the pharmaceutical placebo (*n* = 250, 12.4%) with women who received sham acupuncture and the pharmaceutical placebo (*n* = 250, 15.6%) [risk difference: − 2.9 (− 9.6 to 3.9)]. Nor was there any difference when comparing all women who received acupuncture regardless of whether they received clomiphene (*n* = 500, 20%) with all those who received sham acupuncture (*n* = 500, 21%) [risk difference: − 0.4 (− 5.8 to 5.1)].

Cochrane et al. [24] conducted a RCT comparing the effect of acupuncture and lifestyle changes (diet and exercise advice) with lifestyle modification alone on 56 subfertile women. During the 3 months of intervention and 12 months’ follow-up, there was no difference in pregnancy rate for women receiving acupuncture and women receiving lifestyle intervention alone (14.3% vs 10.7%, *p* = 0.992). Twelve months following intervention, ten women receiving acupuncture and eight women receiving lifestyle intervention alone had become pregnant in total (35.7% vs 17.9%, *p* = 0.131).

The effect of homeopathic therapy on conception was assessed in a double-blinded RCT by Wittmann et al. [25]. 84 women were given 60 drops of Mastodynon or placebo to take daily for 3 months with no additional follow-up. Nine women each in the treatment and control groups became pregnant (21.4% vs 23.1%, *p* = 0.494).

Finally, Holt et al. [26] conducted a sham-controlled double-blinded RCT of eight sessions of reflexology on 49 women with anovulation over 10 weeks with 2 months of follow-up. Four women in the treatment group and two women in the control group conceived (19.0% vs 11.1%, *p* = 0.680).

### Studies reporting an effect

Arentz et al. [27] assessed the effect of up to 3 months’ use of two herbal supplements in 122 overweight women with PCOS receiving diet and exercise advice. There was no follow-up. The treatment group received a tablet combining extracts of Glycyrrhiza glabra, Paeonia lactiflora, Cinnamomum verum and Hypericum perforatum and another containing Tribulus terrestris extract, the latter to be taken during follicular phase of the menstrual cycle. Eleven women in the treatment group became pregnant and three in the control group (32.4% vs 8.3%, *p* = 0.012).

In a double-blind placebo-controlled trial, Westphal et al. [34] investigated the effect of 3 months of FertilityBlend™, a nutritional supplement that included herbal extracts, antioxidants, vitamins and minerals, on 30 women who had been trying to conceive for 6–36 months. No follow-up period was reported. Five women, all of whom had received the supplement, became pregnant (33.3% vs 0.0%, *p* = 0.014).

Four trials found evidence for a positive effect of the antioxidants l-carnitine and l-acetyl-carnitine for men with subfertility. Cavallini et al. [28] randomized 325 men to one of three arms. Treatment group 1 received l-carnitine (2 g/day) and l-acetyl-carnitine (1 g/day) with a cinnoxicam suppository (30 mg) every 4 days. Treatment group 2 received the same doses of l-carnitine and l-acetyl-carnitine and a placebo suppository. The control group received oral and suppository placebos. Treatment was for 6 months with 3 months’ further follow-up. Conception rates were significantly higher in treatment group 1 than the control group (21.8% vs 1.7%, *p* < 0.001) and treatment group 2 than the control group (38.0% vs 1.7%, *p* < 0.001). Conception rates in treatment group 2 were also significantly higher than in treatment group 1 (38.0% vs 21.8%, *p* = 0.012).

In two separate placebo-controlled, double-blind, crossover trials Lenzi et al. [29, 30] assessed the effect of antioxidants on sexually active men with sub-fertility. In both trials, treatment was received for 2 months with 2 months’ washout period in between treatments and 2 months’ follow-up afterwards. The first trial recruited 100 men and evaluated l-carnitine (2 g/day). No pregnancies occurred during the control periods, but eight pregnancies occurred during treatment (0.0% vs 9.3%, *p* = 0.004). In a separate trial, 60 men alternated receiving l-carnitine (2 g/day) with l-acetyl-carnitine (1 g/day) or placebo. Four pregnancies occurred during treatment phases and none during placebo phases (13.3% vs 0.0%, *p* = 0.045).

Busetto et al. [31] investigated the effect of 6 months of antioxidants combined with supplements (1000 mg l-carnitine, 725 mg fumarate, 500 mg acetyl-l-carnitine, 20 mg co-enzyme Q10, 90 mg vitamin C, 10 mg zinc, 100 μg folic acid, 1.5 μg B12) on 104 subfertile men in a double-blind placebo-controlled RCT. Ten partners in the therapy group became pregnant compared to two in the control group in up to 6 months’ follow-up (20.4% vs 4.4%, *p* = 0.021).

Razavi et al. [32] evaluated the effect of selenium (200 μg/day) taken for 2 months on 64 women with PCOS in a placebo-controlled RCT with no follow-up. All women received the same dose of metformin over the trial period. Six women taking selenium and one woman taking the placebo became pregnant (18.8% vs 3.1%, *p* = 0.040).

In a RCT by Lim et al. [33] 146 women with PCOS were randomized to 3 months of true or sham acupuncture with 3 months’ follow-up. Five women who had received true acupuncture became pregnant whereas none of the women in the control group became pregnant (5.1% vs 0.0%, *p* = 0.002).

### Miscarriages

Three RCTs from Australia, China and USA evaluated the number of miscarriages in men and women when treated with a CAM (eTable 6). The studies evaluated a biological based therapy (a supplement), a body manipulation practice (acupuncture) and a traditional CAM (homeopathy). No studies demonstrated that treatment with a CAM affected rates of miscarriage.

### Studies reporting no statistically significant effect

Arentz et al. [27] evaluated the use of herbal supplements for up to 3 months in women with PCOS with no follow-up. Of the 11 pregnancies that occurred in the treatment group, four ended in miscarriage, while one out of the three pregnancies arising in the control group ended in miscarriage (36.4% vs 33.3%, *p* = 0.923).

No statistically significant effect of 16 weeks of acupuncture and 10 months of follow-up on miscarriages for women with PCOS was reported by Wu et al. [23]. The proportion of pregnant women who miscarried was similar in the group receiving acupuncture and a placebo (37.3%) and the control group (29.1%). Compared to the control group, the risk difference of miscarriage for women who had acupuncture and placebo was 8.2 (− 9.7 to 26.1).

Westphal et al. [34] reported one of the five pregnancies that arose in the group receiving 3 months without follow-up of a herbal and dietary supplement ended in miscarriage (20%). No pregnancies occurred in the control group and, therefore, no miscarriages.

### Live births

Four RCTs investigated treatment with a CAM on the rates of live births in women (eTable 7). One study each was from Germany, China, Australia and USA. They assessed the effects of a biological based therapy (a supplement), a body manipulation practice (acupuncture) and a traditional CAM (homeopathy). Only one study found evidence of greater live birth rates for women treated with a CAM.

### Studies reporting no statistically significant effect

Bergmann et al. [21] conducted a placebo-controlled, double-blind RCT of 3 months of homeopathic preparation of Agnus castus extract, with 3 months’ follow-up, recruiting 78 women with infrequent or absent menstruation. There were six live births in the treatment group and two in the control group (20.0% vs 6.7%, *p* = 0.129).

Wu et al. [23] provided no evidence of an effect of 16 weeks of acupuncture on live birth rates in a placebo-controlled, blinded, factorial trial of clomiphene and acupuncture in women with PCOS that had 10 months’ follow-up. There was no significant difference in the rate of live births between the group receiving active acupuncture and a placebo tablet and the control group (16.8%) (13.9%; risk difference, − 2.9% [95% CI: − 9.5–3.7%)].

Arentz et al. [27] provided no evidence of an effect of up to 3 months of homeopathic preparation (containing Glycyrrhiza glabra, Peonia lactiflora, Cinnamonum verum, Hypericum perforatum and Tribulus terrestris) on live birth rates. There was no follow-up. There were seven live births in the treatment group and two in the control group (20.6% vs 5.6%, *p* = 0.060).

### Studies reporting an effect

A single study found evidence of a higher rate of live births for women receiving a CAM. Westphal et al. [34] randomized women who had tried unsuccessfully to conceive for 6–36 months to receiving 3 months of a herbal and dietary supplement, or a placebo. Although the follow-up duration was not reported, all four live births occurred in the treatment group, resulting from five pregnancies that occurred during and in the year after the trial.

### Study quality

**Fig. 3:**
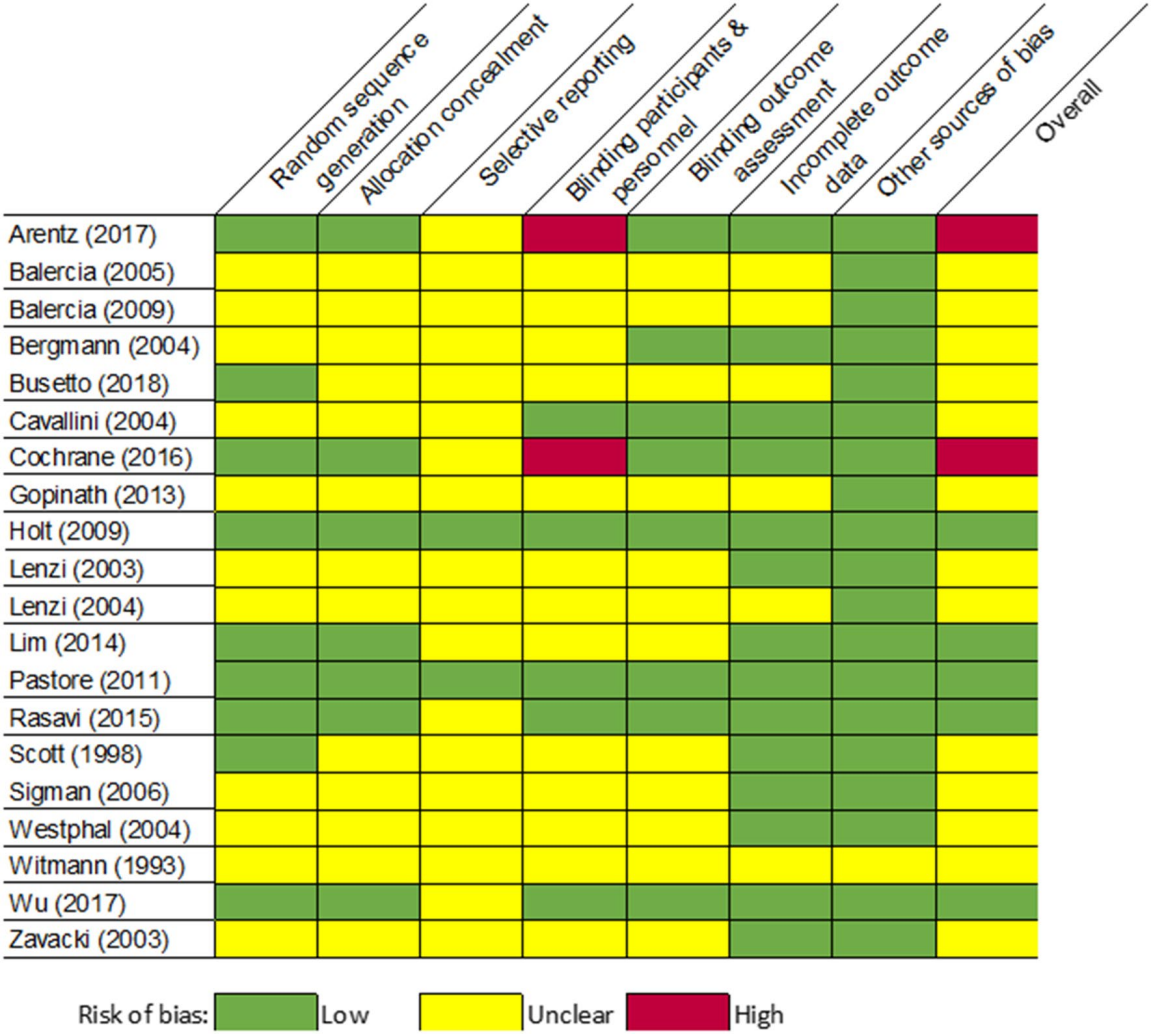
Quality of studies in the review as assessed by the Cochrane Collaboration’s tool

Two out of the 20 included trials demonstrated a high risk of bias, as evaluated using the Cochrane Collaboration tool (Fig. 3, eTable 8). In both trials the high risk of bias related to blinding of participants and personnel. A further 13 trials demonstrated an unclear risk of bias in one or more areas, while 5 trials had a low risk of bias in all areas of study quality.

**Fig. 4:**
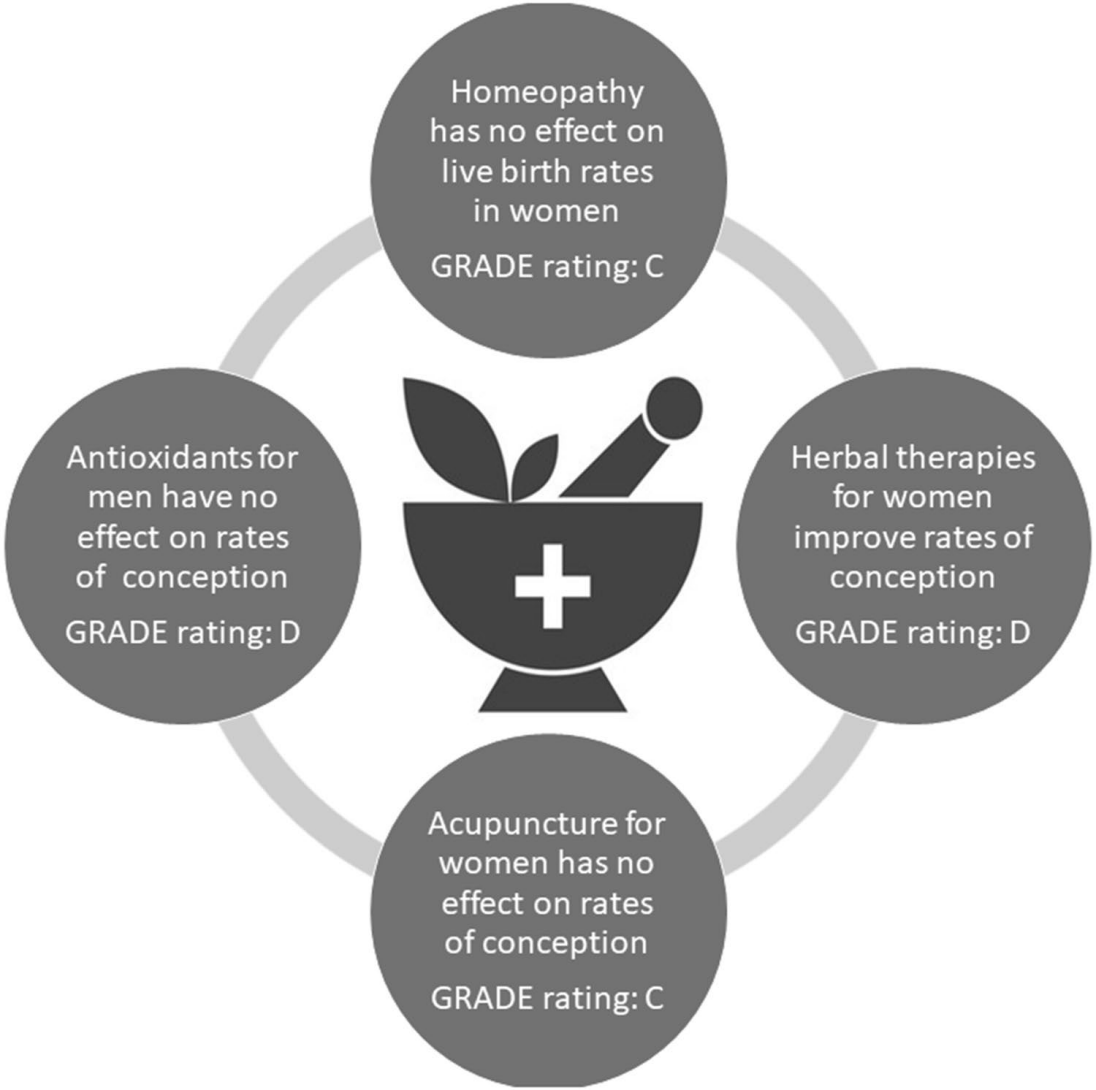
Clinical implications of findings on complementary and alternative medicines and reproductive outcomes. Quality of evidence assessed using Grading of Recommendations Assessment, Development and Evaluation (GRADE): C: low quality of evidence (our confidence in the effect estimate is limited: The true effect may be substantially different from the estimate of the effect). D: very low quality of evidence (out confidence in the effect estimates is severely limited. The true effect is probably markedly different from the estimated effect)

The GRADE approach was used to determine the quality of evidence for exposure–outcome combinations where two or more studies were identified. The grading based on the current review is indicated in Table 2 and Fig. 4. Down-grading due to risk of bias, inconsistency and imprecision resulted in low to very low evidence.

## Discussion

### Summary of the findings

This review, based on data from 20 studies, gives an important overview of the current knowledge on effects of CAM for couples not undergoing ART treatments, related to conception, miscarriage and live births. The majority of studies demonstrated no statistically significant effect of CAM on any of the outcomes, and only eight studies reported an advantageous association. Overall, there was low to very low evidence of any CAM effecting any reproductive outcomes.

### Findings in context of other research into CAM

Although women may avoid conventional and pharmacological therapy during pregnancy due to potential adverse effects [35, 36], CAM are often perceived as natural and safe [37]. However, research is lacking on the efficacy and safety of CAM among pregnant and postpartum women even for commonly used herbal therapies [38]. The most pressing issue from couples’ perspectives is whether the use of a CAM will affect their risk of pregnancy loss or having a live born child. Only 3 out of the 20 identified studies reported the number of miscarriages, and only 4 studies evaluated the probability of having a live born child. Furthermore, no studies evaluated time to pregnancy. This marked lack of research in this area will only compound the limited advice and guidance that caregivers can provide to couples who wish to have a child.

The focus on this review, on CAM use outside of ART treatment, does not negate potential benefits of CAM use for couples seeking to conceive with ART treatment or for women during pregnancy. A previous systematic review of the evidence from trials for CAM during infertility treatment found preliminary evidence of the effectiveness of some CAM interventions [9]. Three or more clinical trials reported results that were consistent with a positive effect of the use of acupuncture, selenium supplementation, weight loss and psychotherapy during infertility treatment. Acupuncture has also been reported to be beneficial as an adjunct to standard analgesia during oocyte retrieval for IVF [9] and to have a beneficial effect on back pain during pregnancy [23].

### Strengths and limitations of the current evidence and directions for future research

To the authors’ knowledge this is the first review of CAM use in couples not undergoing ART treatment. Strengths include the wide range of CAM included in the search terms and multiple databases used in the search, although this limited detailed evaluation of each CAM. Synthesis of the existing knowledge on this topic was challenging due to inconsistent findings between some studies evaluating similar CAM (e.g. Balercia et al. [17] and Cavallini et al. [28]), which may partly reflect differences in the dose, formulation or route of administration of CAM used in studies theoretically evaluating the same exposure. Publication bias may also have impacted the review, although the majority of the studies have small sample sizes and did not report an effect on reproductive outcomes. Furthermore, the majority of included studies are from European populations, which might limit the generalizability of the findings to other populations, and the quality of studies was generally poor. Other important factors such as differences in underlying fertility risk factors in study populations and variability in adjustment levels prevent quantitative synthesis of the studies.

The importance of age in the included studies cannot be overstated. A major determinant of fertility in women, age impacts ovarian reserves and, therefore, the quantity and quality of oocytes available for fertilization. The ability to become pregnant at the age of 34–35 is 14% lower than at the ages of 30–31, which decreases to 50% by the age of 40 [39]. Therefore, the variation in the mean age of female participants in the included studies, from 26 to 34, will have a large effect on the probability of conception and introduced between-study heterogeneity to this review.

Our review should stimulate further research in this area. The aforementioned few studies assessing miscarriage, live birth rates and time to pregnancy is a major gap in the literature. The latter is particularly pertinent; many studies assessed conceptions but neglected to conduct any statistical analyses assessing time to pregnancy. Furthermore, the majority of identified studies assessed biological CAM. In general, the small number of individuals included in most of the studies (number of participants recruited: mean = 137, median = 74) strongly suggests the studies may lack power to detect any significant effect of a CAM and call into question whether significant results reflect chance findings.

To comprehensively investigate the role of CAM, it may be necessary to conduct large population-based studies rather than RCTs. Previous surveys indicate that large numbers of couples use CAM when trying to get pregnant [8] or during pregnancy [40]. Given the small numbers of participants in the trials included in this review (18 studies had < 200 participants), and the relatively short follow-up durations (range: 0–12 months), a cohort study with large numbers of participants followed for a sufficient amount of time (> 5 years) may be more feasible. This would also have the advantage of including women and men with a wide range of ages and, therefore, having the potential to evaluate the effects of a CAM at different ages. A good example of this is the cohort study of over 25,000 individuals evaluating whether use of acupuncture after stroke reduced the risk of pneumonia [41].

## Conclusion

The current review presents a cutting-edge summary of CAM and pregnancy outcomes. However, the findings of this article should be treated cautiously. The quality of many studies included in this review was low, although findings were based on RCTs. Use of CAM should be based on evidence and attention should be paid to negative effects, such as a potential increased risk of miscarriage. Also, it is recommended that medical professionals discuss with patients who approach them with fertility problems about whether they are using any CAM. Although no negative effects on conception were reported, side effects were prevalent. These include diarrhea [20], euphoria [28] and headaches [24].

Overall, the evidence on CAM and pregnancy outcomes for individuals not using ART treatment is not robust due to low study quality and small sample sizes. There is limited support for the role of different CAM, such as herbal therapies in enhancing the chances of conception and subsequent live births.

## Electronic supplementary material

Below is the link to the electronic supplementary material.

Supplementary file1 (DOCX 66 kb)

## Data Availability

All data generated or analyzed during this study are included in this published article and its supplementary information files.

## Abbreviations

ART: Assisted reproductive technology treatment
CAM: Complementary or alternative medicine
PCOS: Polycystic ovary syndrome
