# Supplementary material for: Reproductive outcomes in women and men using complementary and alternative medicine treatment and not receiving artificial reproductive technology: a systematic review
Source: Arch Gynecol Obstet. 2020 Oct 20;303(3):821–35. doi: 10.1007/s00404-020-05836-4 (PMC7960609; doi:10.1007/s00404-020-05836-4)
Supplement: Supplementary file 1 — Supplementary file1 (DOCX 66 kb) [file 404_2020_5836_MOESM1_ESM.docx]

**Reproductive outcomes in women and men using complementary and alternative medicine treatment: a systematic review**

**Supplementary Material**

[eTable 1: PRISMA checklist 2](#_Toc39898152)

[eTable 2: Search terms used in PubMed 7](#_Toc39898153)

[eTable 3: List of complementary and alternative medicines included in the search terms. 16](#_Toc39898154)

[eTable 4: Further details of studies included in the review. 18](#_Toc39898155)

[eTable 5: Results of studies evaluating conception in relation to a complementary or alternative medicine. 27](#_Toc39898156)

[eTable 6: Results of studies evaluating miscarriages in relation to a complementary or alternative medicine. 37](#_Toc39898157)

[eTable 7: Results of studies evaluating live-births in relation to a complementary or alternative medicine. 39](#_Toc39898158)

[eTable 8: Quality of studies in the review as assessed by the Cochrane Collaboration’s tool. 42](#_Toc39898159)

## eTable 1: PRISMA checklist

| **Section/topic** | **#** | **Checklist item** | **Reported on page #** |
| --- | --- | --- | --- |
| **TITLE** | | |  |
| Title | 1 | Identify the report as a systematic review, meta-analysis, or both. | 1,2,3 |
| **ABSTRACT** | | |  |
| Structured summary | 2 | Provide a structured summary including, as applicable: background; objectives; data sources; study eligibility criteria, participants, and interventions; study appraisal and synthesis methods; results; limitations; conclusions and implications of key findings; systematic review registration number. | 2 |
| **INTRODUCTION** | | |  |
| Rationale | 3 | Describe the rationale for the review in the context of what is already known. | 3 |
| Objectives | 4 | Provide an explicit statement of questions being addressed with reference to participants, interventions, comparisons, outcomes, and study design (PICOS). | 3, 4 |
| **METHODS** | | |  |
| Protocol and registration | 5 | Indicate if a review protocol exists, if and where it can be accessed (e.g., Web address), and, if available, provide registration information including registration number. | 2,4 |
| Eligibility criteria | 6 | Specify study characteristics (e.g., PICOS, length of follow-up) and report characteristics (e.g., years considered, language, publication status) used as criteria for eligibility, giving rationale. | 3,4, 5 |
| Information sources | 7 | Describe all information sources (e.g., databases with dates of coverage, contact with study authors to identify additional studies) in the search and date last searched. | 4 |
| Search | 8 | Present full electronic search strategy for at least one database, including any limits used, such that it could be repeated. | eTable 2 |
| Study selection | 9 | State the process for selecting studies (i.e., screening, eligibility, included in systematic review, and, if applicable, included in the meta-analysis). | 4, 5 |
| Data collection process | 10 | Describe method of data extraction from reports (e.g., piloted forms, independently, in duplicate) and any processes for obtaining and confirming data from investigators. | 5 |
| Data items | 11 | List and define all variables for which data were sought (e.g., PICOS, funding sources) and any assumptions and simplifications made. | 5 |
| Risk of bias in individual studies | 12 | Describe methods used for assessing risk of bias of individual studies (including specification of whether this was done at the study or outcome level), and how this information is to be used in any data synthesis. | 6 |
| Summary measures | 13 | State the principal summary measures (e.g., risk ratio, difference in means). | 5,6 |
| Synthesis of results | 14 | Describe the methods of handling data and combining results of studies, if done, including measures of consistency (e.g., I2) for each meta-analysis. | NA |
| Additional analyses | 16 | Describe methods of additional analyses (e.g., sensitivity or subgroup analyses, meta-regression), if done, indicating which were pre-specified. | 5,6 |
| **RESULTS** | | |  |
| Study selection | 17 | Give numbers of studies screened, assessed for eligibility, and included in the review, with reasons for exclusions at each stage, ideally with a flow diagram. | Figure 1, 7 |
| Study characteristics | 18 | For each study, present characteristics for which data were extracted (e.g., study size, PICOS, follow-up period) and provide the citations. | Table 1, eTable 4 |
| Risk of bias within studies | 19 | Present data on risk of bias of each study and, if available, any outcome level assessment (see item 12). | eTable 8 |
| Results of individual studies | 20 | For all outcomes considered (benefits or harms), present, for each study: (a) simple summary data for each intervention group (b) effect estimates and confidence intervals, ideally with a forest plot. | eTables 5-6 |
| Synthesis of results | 21 | Present results of each meta-analysis done, including confidence intervals and measures of consistency. | N/A |
| Risk of bias across studies | 22 | Present results of any assessment of risk of bias across studies (see Item 15). | Figure 2, Table 2 |
| Additional analysis | 23 | Give results of additional analyses, if done (e.g., sensitivity or subgroup analyses, meta-regression [see Item 16]). | eTable 5-7 |
| **DISCUSSION** | | |  |
| Summary of evidence | 24 | Summarize the main findings including the strength of evidence for each main outcome; consider their relevance to key groups (e.g., healthcare providers, users, and policy makers). | 15 |
| Limitations | 25 | Discuss limitations at study and outcome level (e.g., risk of bias), and at review-level (e.g., incomplete retrieval of identified research, reporting bias). | 16 |
| Conclusions | 26 | Provide a general interpretation of the results in the context of other evidence, and implications for future research. | 17 |
| **FUNDING** | | |  |
| Funding | 27 | Describe sources of funding for the systematic review and other support (e.g., supply of data); role of funders for the systematic review. | 18 |

## eTable 2: Search terms used in PubMed

| Population | ("humans"[MeSH Terms]) AND |
| --- | --- |
| Complementary and alternative medicine | (“pelvic physical therap*”[All Fields] OR “pelvic physiotherap*”[All Fields] OR “hypnosi*”[All Fields] OR "Hypnosis"[Mesh] OR “hypnotherapy*”[All Fields] OR “Mesmerism”[All Fields] OR “spiritual heal*”[All Fields] OR "Spiritual Therap*"[All Fields] OR "Spiritual Therapies"[Mesh] OR “Robitussin*”[All Fields] OR “Sage*”[All Fields] OR "Salvia officinalis"[Mesh] OR "Salvia"[All Fields] OR “Melatonin*”[All Fields] OR “ N-acetyl-5-methoxy tryptamine”[All Fields] OR "Melatonin"[Mesh] OR “*Resveratrol*”[All fields] OR "resveratrol" [Supplementary Concept] OR “Royal Jell*”[All Fields] OR "royal jelly" [Supplementary Concept] OR “American Ginseng*”[All Fields] OR “Panax quinquefolius” [All Fields] “Panacis quinquefolis”[All Fields] OR “huaqishen”[All Fields] OR “west ocean ginseng*"[All Fields] OR “Flower Flog ginseng*”[All Fields] OR "Panax"[Mesh] OR “Cordycep*”[All Fields] OR "Cordyceps"[Mesh] OR “Epimedium*”[All Fields] OR “horny goat weed*”[All Fields] OR "Epimedium"[Mesh] OR “Ginkgo biloba*”[All Fields] OR “Maidenhair Tree*”[All Fields] OR “Ginkgophyta*”[All Fields] OR “Ginkgo biloba"[Mesh] OR “Goji berr*”[All Fields] OR “Lycium barbarum” [All Fields] OR “Lyclum chinense”[All Fields] OR "Lycium"[Mesh] OR “Wolfberr*”[All Fields] OR “Saw Palmetto *”[All Fields] OR “Serenoa repens”[All Fields] OR "saw palmetto extract" [Supplementary Concept] OR "Serenoa"[Mesh] OR “Permixon*”[All Fields] OR “CoQ10“[All Fields] OR “ubiquinol*“[All Fields] OR “coenzyme Q10“[All Fields] OR “co-enzyme Q10”[All Fields] OR “ubiquinone*”[All Fields] OR "coenzyme Q10" [Supplementary Concept] OR"ubiquinol-10" [Supplementary Concept] OR “Glutathione*”[All Fields] OR "Glutathione"[Mesh] OR “Alpha-lipoic acid*”[All Fields] OR “R-alpha-lipoic acid*”[All Fields] OR “*alpha-lipoic acid*”[All Fields] OR “alpha lipoic acid*”[All Fields] OR "Thioctic Acid*"[All Fields] OR "Thioctic Acid*”[Mesh] OR "alpha-lipoic acid, 4-aminobenzoic acid, aniline, benfotiamine, thioctic Acid, vitamin E drug combination" [Supplementary Concept] OR “Myo-inositol*”[All Fields] OR “Myoinositol*”[All Fields] OR "Inositol*"[All Fields] OR "Inositol"[Mesh] OR “Progesterone cream*”[All Fields] OR “DHEA*”[All Fields] OR “dehydroepiandrosterone*”[All Fields] OR "Dehydroepiandrosterone"[Mesh] OR “Chiropody”[All Fields] OR “chiropractic”[MeSH Terms] OR “chiropractic*”[All Fields] OR “Essential oil*”[All Fields] OR “roman chamomile*”[All Fields] OR “thyme*”[All Fields] OR “ylang ylang*”[All Fields] OR “ylang-ylang*”[All Fields] OR "Oils, Volatile"[Mesh] OR "Volatile Oil*"[All Fields] OR "Chamaemelum"[Mesh] OR "Thymus Plant"[Mesh] OR "Cananga"[Mesh] OR “Tribulus”[All Fields] OR “Puncture Vine*”[All Fields] OR “goathead*”[All Fields] OR "Tribulus"[Mesh] OR “Rehmannia”[All Fields] OR “Chinese Foxglove*”[All Fields] OR "Rehmannia"[Mesh] OR “Omega fatty acid*”[All Fields] OR “*PUFA*”[All Fields] OR “Polyunsaturated fatty acid*”[All Fields] OR “Omega-3”[All Fields] OR “Omega-6”[All Fields] OR “Omega-9”[All Fields] OR “alpha-linolenic acid*”[All Fields] OR “alpha linolenic acid*”[All Fields] OR “eicosapentaenoic acid*”[All Fields] OR “docosahexaenoic acid*”[All Fields] OR “α-linolenic acid*”[All Fields] OR “fish oil*”[All Fields] OR “Linoleic acid*”[All Fields] OR “oleic acid*”[All Fields] OR “elaidic acid*”[All Fields] OR “gondoic acid*”[All Fields] OR “mead acid*”[All Fields] OR “erucic acid*”[All Fields] OR “nervonic acid*”[All Fields] OR “ximenic acid*”[All Fields] OR “Gamma-linolenic acid*”[All Fields] OR “Calendic acid*”[All Fields] OR “Eicosadienoic acid*”[All Fields] OR “Dihomo-gamma-linolenic acid*”[All Fields] OR “Arachidonic acid*”[All Fields] OR “Docosadienoic acid*”[All Fields] OR “Adrenic acid*”[All Fields] OR “Osbond acid*”[All Fields] OR “Tetracosatetraenoic acid*”[All Fields] OR “Tetracosapentaenoic acid*”[All Fields] OR "Fatty Acids, Omega-3"[Mesh] OR "Fatty Acids, Omega-6"[Mesh] OR “Black Cohosh*”[All Fields] OR “Cimicifuga racemosa”[All Fields] OR “Actaea racemosa”[All Fields] OR "Cimicifuga"[Mesh] OR “Mugwort*”[All Fields] OR “Artemisia vulgaris”[All Fields] OR "Artemisia"[Mesh] OR “Parsley*”[All Fields] OR “Petroselinum crispum”[All Fields] OR "Petroselinum"[Mesh] OR “Agnus castus”[All Fields] OR “Vitex”[All Fields] OR “chasteberr*”[All Fields] OR “Chaste Tree Berr*”[All Fields] OR “Chaste$tree*”[All Fields] OR “Vitex agnus$castus”[All Fields] OR "Vitex"[Mesh] OR “Evening primrose oil*”[All Fields] OR “Oenothera biennis”[All Fields] OR “evening star*”[All Fields] OR “sun drop*”[All Fields] OR “German rampion*”[All Fields] OR “hog$weed*”[All Fields] OR “King's cure-all*”[All Fields] OR “fever$plant*”[All Fields] OR "evening primrose oil" [Supplementary Concept] OR “marshmallow root*”[All Fields] OR “Althea officinalis”[All Fields] OR “marsh-mallow*”[All Fields] OR “marsh$mallow*”[All Fields] OR "Althaea"[Mesh] OR “Oregon Grape*”[All Fields] OR “Mahonia aquifolium*”[All Fields] OR “Oregon-Grape*”[All Fields] OR “barberr*”[All Fields] OR "Mahonia"[Mesh] OR “Flax$”[All Fields] OR “Linum usitatissimum”[All Fields] OR “linseed*”[All Fields] OR "Flax"[Mesh] OR “Licorice*”[All Fields] OR “Glycyrrhiza glabra”[All Fields] OR “Liquorice*”[All Fields] OR "Glycyrrhiza"[Mesh] OR “Sesame seed*”[All Fields] OR “Sesamum indicum”[All Fields] OR "oleosin protein, Sesamum indicum" [Supplementary Concept] OR “Shatavari*”[All Fields] OR “Asparagus racemosus”[All Fields] OR “shatamull*”[All Fields] OR “satavar*”[All Fields] OR “Cranesbill*”[All Fields] OR “Geranium maculatum”[All Fields] OR “Geranium*”[All Fields] OR "Geranium"[Mesh] OR “Cinnamon*”[All Fields] OR “Cinnamomum verum”[All Fields] OR “Cinnamomum zeylanicum”[All Fields] OR "Cinnamomum zeylanicum"[Mesh] OR “Liferoot*”[All Fields] OR “Senecio aureus”[All Fields] OR “Packera aurea”[All Fields] OR “ragwort*”[All Fields] OR “golden ragwort*”[All Fields] OR “groundsel*”[All Fields] OR “squaw weed*”[All Fields] OR “life root*”[All Fields] OR “golden Senecio*”[All Fields] OR “uncum*”[All Fields] OR “waw weed*”[All Fields] OR “false valerian*”[All Fields] OR “cough weed*”[All Fields] OR “female regulator*”[All Fields] OR “cocash weed*”[All Fields] OR “ragweed*”[All Fields] OR “staggerwort*”[All Fields] OR “St. James wort*”[All Fields] OR "Senecio"[Mesh] OR “Maca *”[All Fields] OR “Lepidium meyenii”[All Fields] OR “maca$maca*”[All Fields] OR “maino*”[All Fields] OR “ayak chichira*”[All Fields] OR “ayak willku*”[All Fields] OR "Lepidium"[Mesh] OR “Nettle*”[All Fields] OR “Urtica dioica”[All Fields] OR "Urtica dioica"[Mesh] OR “blue periwinkle*”[All Fields] OR “Vinca major”[All Fields] OR “bigleaf periwinkle*”[All Fields] OR “large periwinkle*”[All Fields] OR “greater periwinkle*”[All Fields] OR "Vinca"[Mesh] OR “Seaweed*”[All Fields] OR “Dulse*”[All Fields] OR “Kombu*”[All Fields] OR “Wakame*”[All Fields] OR "Undaria"[Mesh] OR "Laminaria"[Mesh] OR "Seaweed"[Mesh] OR “Shepherd*s purse*”[All Fields] OR “Capsella bursa$pastoris”[All Fields] OR "Capsella"[Mesh] OR “White peon*”[All Fields] OR “Paeonia lactiflora”[All Fields] OR “Chinese peon*”[All Fields] OR “common garden peon*”[All Fields] OR "Paeonia"[Mesh] OR “Yarrow*”[All Fields] OR “Achillea millefolium”[All Fields] OR "Achillea"[Mesh] OR “Yellow Dock*”[All Fields] OR “Rumex crispus”[All Fields] OR “curly dock*”[All Fields] OR “curled dock*”[All Fields] OR “French sorrel*”[All Fields] OR “Canaigre”[All Fields] OR "Rumex"[Mesh] OR “Black haw*”[All Fields] OR “Viburnum prunifolium”[All Fields] OR “blackhaw*”[All Fields] OR “sweet haw*”[All Fields] OR “stag bush*”[All Fields] OR "Viburnum"[Mesh] OR “Cramp bark*”[All Fields] OR “Viburnum opulus”[All Fields] OR “guilder$rose*”[All Fields] OR “water elder*”[All Fields] OR “snowball tree*”[All Fields] OR “European cranberrybush*”[All Fields] OR “False Unicorn”[All Fields] OR “Chamaelirium luteum”[All Fields] OR “blazing-star”[All Fields] OR “devil's bit”[All Fields] OR “fairy wand”[All Fields] OR “helonias”[All Fields] OR “Partridge Berry”[All Fields] OR “Mitchella repens”[All Fields] OR “squaw vine”[All Fields] OR “partridgeberry”[All Fields] OR “squaw berry”[All Fields] OR “two-eyed berry”[All Fields] OR “running fox”[All Fields] OR “Noon kie oo nah yeah”[All Fields] OR “Wild Yam*”[All Fields] OR “Dioscorea villosa”[All Fields] OR “Chinese Yam*” OR "Dioscorea"[Mesh] OR “Bee Pollen*”[All Fields] OR “Propolis”[All Fields] OR "Propolis"[Mesh] OR “Burdock*”[All Fields] OR “Arctium lappa”[All Fields] OR “gobō”[All Fields] OR “lappa”[All Fields] OR “beggar's button*”[All Fields] OR “thorny burr*”[All Fields] OR “happy major*”[All Fields] OR “Arctium"[Mesh] OR “Castor Oil*”[All Fields] OR “Ricinus communis”[All Fields] OR “castorbean*”[All Fields] OR “castor-oil-plant*”[All Fields] OR “castor oil plant*”[All Fields] OR "Castor Oil"[Mesh] OR “Fo$ti”[All Fields] OR “Polygonum multiflorum”[All Fields] OR “He Shou Wu”[All Fields] OR “Ho Shou Wu”[All Fields] OR “tuber fleeceflower*”[All Fields] OR “Chinese knotweed”[All Fields] OR "Fallopia multiflora"[Mesh] OR “Ginger*”[All Fields] OR “Zingiber officinale”[All Fields] OR "Ginger"[Mesh] OR “Lemon Balm*”[All Fields] OR “Melissa officinalis”[All Fields] OR “common balm*”[All Fields] OR “balm mint*”[All Fields] OR "Melissa"[Mesh] OR “Motherwort*”[All Fields] OR “Leonurus cardiaca”[All Fields] OR “throw$wort*”[All Fields] OR “lion's ear*”[All Fields] OR “lion's tail*”[All Fields] OR "Leonurus"[Mesh] OR “Oat straw*”[All Fields] OR “Milky Oat*”[All Fields] OR “Avena sativa”[All Fields] OR “oatstraw*”[All Fields] OR "Avena"[Mesh] OR “Alfalfa”[All Fields] OR “Medicago sativa”[All Fields] OR “lucerne”[All Fields] OR "Medicago sativa"[Mesh] OR “Ashwagandha”[All Fields] OR “Withania*”[All Fields] OR “Indian ginseng*”[All Fields] OR “poison gooseberry*”[All Fields] OR “winter cherr*”[All Fields] OR "Withania"[Mesh] OR “Dandelion*”[All Fields] OR “Taraxacum officinale”[All Fields] OR "Taraxacum"[Mesh] OR “Dong Quai”[All Fields] OR “Angelica sinensis”[All Fields] OR “female ginseng*”[All Fields] OR "Angelica sinensis"[Mesh] OR “Hibiscus”[All Fields] OR “Hibiscus sabdariffa”[All Fields] OR "Hibiscus"[Mesh] OR “Milk Thistle*”[All Fields] OR “Silybum marianum”[All Fields] OR “cardus marianus”[All Fields] OR “blessed milk thistle*”[All Fields] OR “Marian thistle*”[All Fields] OR “Mary thistle*”[All Fields] OR “Saint Mary's thistle*”[All Fields] OR “Mediterranean milk thistle*”[All Fields] OR “variegated thistle*”[All Fields] OR “Scotch thistle*”[All Fields] OR "Milk Thistle"[Mesh] OR “Red Clover”[All Fields] OR “Trifolium pratense”[All Fields] OR "Trifolium"[Mesh] OR “Red Raspberry lea*”[All Fields] OR “Rubus idaeus”[All Fields] OR "Rubus"[Mesh] OR “Schisandra”[All Fields] OR “Schisandra chinensis”[All Fields] OR “magnolia-vine*”[All Fields] OR “magnolia berr*”[All Fields] OR “five-flavo$r-fruit*”[All Fields] OR "Schisandra"[Mesh] OR “Eleuthero”[All Fields] OR “Eleutherococcus senticosus”[All Fields] OR “Siberian ginseng*”[All Fields] OR “ciwujia”[All Fields] OR "Eleutherococcus"[Mesh] OR “Chamomile*”[All Fields] OR “Matricaria recutita”[All Fields] OR “scented mayweed*”[All Fields] OR "Eleutherococcus"[Mesh] OR “Linden*”[All Fields] OR “Tilia platyphyllos”[All Fields] OR “Tilia cordata”[All Fields] OR "Tilia"[Mesh] OR “Reishi mushroom”[All Fields] OR “Ganoderma lucidum”[All Fields] OR “lingzhi mushroom”[All Fields] OR "Reishi"[Mesh] OR “Borage seed Oil”[All Fields] OR “Borago officinalis”[All Fields] OR “starflower*”[All Fields] OR "Borago"[Mesh] OR “Blue Cohosh*”[All Fields] OR “Caulophyllum thalictroides”[All Fields] OR “squaw root*”[All Fields] OR “papoose root*”[All Fields] OR "Caulophyllum"[Mesh] OR “Mindfullness”[All Fields] OR “mindfulness”[MeSH Terms] OR “Acupuncture*”[All Fields] OR “acupuncture”[MeSH Terms] OR “Aromatherapy”[All Fields] OR “aromatherapy”[MeSH Terms] OR “Alternative medicine”[All Fields] OR “Complementary therapies”[All Fields] OR “Complementary Therapies”[MeSH Terms] OR “Therapeutic touch”[All Fields] OR “reiki”[All Fields] OR “laying-on-of-hands”[All Fields] OR “therapeutic touch”[MeSH Terms] OR “Healing touch”[All Fields] OR “relaxation therapy”[MeSH Terms] OR “relaxation therap*”[All Fields] OR “relaxation technique*”[All Fields] OR OR “homeopathy”[MeSH Terms] OR “homeopath*”[All Fields] OR “Massage”[MeSH Terms] OR “Massage*”[All Fields]) “Acupressure”[All Fields] OR “Shiatsu”[All Fields] OR “Zhi Ya”[All Fields] OR “Chih Ya”[All Fields] OR “Shiatzu”[All Fields] OR “Acupressure"[Mesh] OR Meridian*”[All Fields] OR “Ching Lo”[All Fields] OR “Jing Luo”[All Fields] OR “Meridians"[Mesh] OR “Acupoint*”[All Fields] OR “Acupuncture Point*”[All Fields] OR "Acupuncture Points"[Mesh] OR “Ayurved*”[All Fields] OR “Ayurvedic medicine”[All Fields] OR “Hindu Medicine”[All Fields] OR “Siddha Medicine”[All Fields] OR “Medicine, Ayurvedic"[Mesh] “Bioelectromagneti*”[All Fields] OR “Physiological Feedback”[All Fields] OR “Myofeedback”[All Fields] OR “Biofeedback*”[All Fields] OR “Neurofeedback*”[All Fields] OR “Brainwave Feedback*”[All Fields] OR “EEG Feedback*”[All Fields] OR “Electromyography Feedback*”[All Fields] OR "Neurofeedback"[Mesh] OR "Biofeedback, Psychology"[Mesh] OR “energy healing*”[All Fields] OR “Chakra”[All Fields] OR “Ethnobotan*”[All Fields] OR “Ethnobotany"[Mesh] OR “guided imag*”[All Fields] OR “Directed Reverie Therap*”[All Fields] OR "Imagery (Psychotherapy)"[Mesh] OR “Herb*”[All Fields] OR “Medicinal plant*”[All Fields] OR “Medicinal Herb*”[All Fields] OR “Healing Plant*”[All Fields] OR “Pharmaceutical Plant*”[All Fields] OR "Plants, Medicinal"[Mesh] OR “Holistic*”[All Fields] OR “hops flower*”[All Fields] OR “Humulus lupulus”[All Fields] OR “seed cone*”[All Fields] OR “strobiles”[All Fields] OR "Humulus"[Mesh] OR “integrative medicine”[All Fields] OR "Integrative Medicine"[Mesh] OR “Isoflavone*”[All Fields] OR “Homoisoflavone*”[All Fields] OR “Isoflavones"[Mesh] OR “soy*”[All Fields] OR “Glycine max”[All Fields] OR “Soybeans"[Mesh] OR “Macrobiotic”[All Fields] OR "Diet, Macrobiotic"[Mesh] OR “music therap*”[All Fields] OR “dietary supplement*”[All Fields] OR “Nutraceutical”[All Fields] OR “Nutriceutical”[All Fields] OR “Herbal supplement*”[All Fields] OR “Food supplement*”[All Fields] OR "Dietary Supplements"[Mesh] OR “probiotic*”[All Fields] OR “Probiotics"[Mesh] OR “qi”[All Fields] OR “life force”[All Fields] OR “vital energy*”[All Fields] OR “Life Breath”[All Fields] OR “Life-breath”[All Fields] OR “Ch’i”[All Fields] OR "Qi"[Mesh] OR “Shaman”[All Fields] OR “Shamanism”[All Fields] OR “Shamanism"[Mesh] OR “spiritual healing”[All Fields] OR “spiritual therap*”[All Fields] OR “Spiritual Therapies"[Mesh] OR “chinese medicine*”[All Fields] OR “Chung I Hsueh”[All Fields] OR “Zhong Yi Xue”[All Fields] OR “Tibetan Medicine*”[All Fields] OR “Chinese Herbal Drug*”[All Fields] OR “Chinese Herbal therap*”[All Fields] OR “Yin Deficienc*”[All Fields] OR “Yin Xu”[All Fields] OR “Yin Hsu”[All Fields] OR “Yinxu”[All Fields] OR “Yang Deficienc*”[All Fields] OR “Yangxu”[All Fields] OR “Yang Xu”[All Fields] OR “Yang Hsu”[All Fields] OR "Medicine, Chinese Traditional"[Mesh] OR "Medicine, Tibetan Traditional"[Mesh] OR "Yang Deficiency"[Mesh] OR "Yin Deficiency"[Mesh] OR “electroacupuncture”[All Fields] OR “Electric* Nerve Stimulation*”[All Fields] OR “Therapeutic Nerve Stimulation*”[All Fields] OR “Electrotherap*”[All Fields] OR “Electric* Stimulation*”[All Fields] OR "Electroacupuncture"[Mesh] OR "Transcutaneous Electric Nerve Stimulation"[Mesh] OR "Electric Stimulation Therapy"[Mesh] |
| Outcomes | (“Pregnan*”[All Fields] OR “conception*”[All Fields] OR “conceiv*”[All Fields] OR “*gravid*”[All Fields] OR “*parous”[All Fields] OR “*parity”[All Fields] OR “gestation*”[All Fields] OR “prenatal*”[All Fields] OR “fertiliz*”[All Fields] OR “Impregnat*”[All Fields] OR “Reproduc*”[All Fields] OR “Procreat*”[All Fields] OR “Breed*”[All Fields] OR “fertilis*”[All Fields] OR “Fertilization"[Mesh] OR "Pregnancy"[Mesh] OR "Gravidity"[Mesh] OR "Parity"[Mesh] OR "Reproduction"[Mesh] OR "Breeding"[Mesh] OR “Pregnancy Loss*”[All Fields] OR “spontaneous abortion*”[All Fields] OR “Miscarriage*”[all fields] OR “tubal abortion*”[All Fields] OR “embryo loss*”[All Fields] OR "Abortion, Spontaneous"[Mesh] OR “habitual abortion*”[All Fields] OR “recurrent abortion*”[All Fields] OR "Abortion, Habitual"[Mesh] OR “threatened abortion*”[All Fields] OR “threatened miscarriage*”[All Fields] OR "Abortion, Threatened"[Mesh] OR “incomplete abortion*”[All Fields] OR “incomplete miscarriage*”[All Fields] OR "Abortion, Incomplete"[Mesh] OR “Subfertil*”[All Fields] OR “Infertil*”[All Fields] OR "Infertility"[Mesh]) AND |
| Study design | (“longitudinal studies"[MeSH Terms] OR “longitudinal study”[All Fields] OR “longitudinal studies”[All Fields] OR "prospective"[All Fields] OR “cohort”[All Fields] OR “cohorts”[All Fields] OR “follow up”[All Fields] OR “follow-up”[All Fields] OR "Clinical Trials as Topic"[Mesh] OR “Clinical trial”[All Fields] OR “Clinical trials”[All Fields] OR "Randomized Controlled Trial" [Publication Type] OR “RCT”[All Fields] OR “Randomised Controlled Trial”[All Fields] OR “Randomized Controlled Trial”[All Fields] OR "Epidemiology"[Mesh] OR "Epidemiology"[All Fields] OR "Epidemiological"[All Fields] OR "Retrospective Studies"[Mesh] OR "Retrospective"[All Fields] OR “prospective”[All Fields] OR "Cross-Sectional Studies"[Mesh] OR "Cross-Sectional"[All fields] OR "Cross Sectional"[All fields]) |

## eTable 3: List of complementary and alternative medicines included in the search terms.

| Acupressure | Chamomile | Ganoderma lucidum | Melatonin | Robitussin |
| --- | --- | --- | --- | --- |
| Acupuncture | Chinese Traditional medicine | Geranium | Melissa officinalis | Royal Jelly |
| Agnus castus | Chiropody | Ginger | Mindfullness | Sage |
| Alpha-lipoic acid | Cinnamon | Ginkgo biloba | Mugwort | Saw Palmetto |
| Alternative medicine | Coenzyme Q10 | Glutathione | Music Therapy | Schisandra chinensis |
| American Ginseng | Complementary Therapies | Goji berries | Myo-inositol | Seaweed, including Dulse, Kombu, & Wakame |
| Arctium lappa | Cordyceps | Guided Imagery | Nettle | Sesame |
| Aromatherapy | Cramp bark | Hibiscus sabdariffa | Omega 3 or 6 fatty acids | Shamanism |
| Asparagus racemosus | Curly Dock | Homeopathy | Oregon Grape | Shepherd’s purse |
| Avena sativa | Dehydroepiandrosterone | Humulus lupulus | Parsley | Silybum marianum |
| Ayurvedic medicine | Dietary or herbal supplements | Hypnosis or hypnotherapy | Partridge Berry | Soy |
| Bee Pollen | Dioscorea polystachya | Integrative Medicine | Pelvic physical therapy | Spiritual healing |
| Bioelectromagnetism | Dioscorea villosa | Isoflavone | Polygonum multiflorum | St. James wort |
| Biofeedback | Dong Quai | Leonurus cardiaca | Probiotics | Taraxacum officinale |
| Black Cohosh | Electroacupuncture | Licorice | Progesterone cream | Therapeutic touch |
| Blue Cohosh | Eleutherococcus senticosus | Linseed | Qi | Tibetan Traditional Medicine |
| Blue Periwinkle | Energy Healing | Maca root | Ragwort | Tilia platyphyllos & Tilia cordata |
| Borage seed Oil | Epimedium | Macrobiotic diet | Red Raspberry Leaves | Tribulus |
| Canaigre | Essential oils, including Thyme, ylang ylang, & chamomile | Marshmallow Root | Rehmannia | Trifolium pretense |
| Cananga | Ethnobotany | Massage | Reishi mushroom | Viburnum prunifolium |
| Castor Oil | Evening primrose oil | Medicago sativa | Relaxation Therapy | White peony |
| Chamaelirium luteum | French sorrel | Medicinal plant | Resveratrol | Withania somnifera |
|  |  |  |  | Yarrow |

## eTable 4: Further details of studies included in the review.

| Study, year | Lost to follow-up (%) | Recruitment process | Recruitment criteria |
| --- | --- | --- | --- |
| Arentz 2017 | 11.50% | Facebook ads, referrals from healthcare providers | Women (18-44 years) with PCOS confirmed using Rotterdam criteria and BMI >24.5kg/m^2^. No taking: oestrogens, progestogens, SSRIs, SNRIs, tetracyclic antidepressants, noradrenergic and selective serotonin reuptake inhibitors, MAO inhibitors, melatonergic antidepressants. |
| Balercia 2005 | 1.7% | Recruited from the Andrology Unit of Marche | Men (20-40 years) with idiopathic asthenozoospermia. Infertility >2 years despite regular sexual intercourse. No female signs of sterility (test progesterone levels to check ovulation, ultrasound of uterus etc). Sperm had normal rheologic characteristics, volume & pH, sperm count >20*10^6mL, normal sperm motility, normal sperm morphological features. seminar WBC must be <1*10^6mL, negative sperm culture, negative chlamydia and mycoplasma urealyticum detection, normal serum levels of gonadotropins, no infectious genital disease, no anatomical abnormalities, no systemic diseases, no treatment with other drugs 3 months before study, no smoking, alcohol, recreational drug use of occupational chemical exposure |
| Balercia 2009 | 8.3% | Recruited from the Andrology Unit of Marche | Men aged 20-40 with idiopathic asthenozoospermia. primary infertility >2 years despite regular sexual intercourse. No female signs of sterility (test progesterone levels to check ovulation, ultrasound of uterus etc). Sperm had normal rheological characteristics, volume & pH, sperm count >20x10^6^mL, normal sperm motility, normal sperm morphological features. seminal WBC <1x10^6^mL, negative sperm culture, negative chlamydia and mycoplasma urealyticum detection, normal serum levels of gonadotropins, no infectious genital disease, no anatomical abnormalities, no systemic diseases, no treatment with other drugs 3 months before study, no smoking, alcohol, recreational drug use of occupational chemical exposure |
| Bergmann 2004 | 14.10% | Recruited from the Women's Health Clinic at Heidelberg University | Women with oligomenorrhoea or amenorrhoea, partner has mobile sperm in the Sims-Huhner postcoital test, at least 1 patent fallopian tube. Exclusion: women with hypothalamic pituitary axis dysregulation (GnRH test was performed two times), pregnancy, pathological spermiogram (WHO 1992), use of hormone therapy in the last 3 months before, (except those who were on l-thyroxine therapy for >3 months), severe acute or chronic disease, alcohol abuse or intolerance. |
| Busetto 2018 | 9.6% | Recruited from the Andrology Unit of Rome Sapienza University | Men aged 18-50 with or without variocele that have oligo/astheno/teratozoospermia, been infertile for >1 year, no other fertility related diseases. Exclusion: known hypersensitivity to any treatment compounds, history of undescended testes or cancer, endocrine disorders, history of post-pubertal mumps, genitourinary surgery, obstructive azoospermia obstructive pathology of urogenital system, autoimmune disease, cystic fibrosis, history of taking any therapy affecting fertility in last 3 months, excessive consumption of alcohol, use of illicit drugs, positive HIV serology, following any special diet, any condition which in the opinion of the investigator might put the subject at risk by participating, involvement in other trials. other causes of infertility. |
| Cavallini 2004 | 14.5% | unstated, possibly recruited from patients attending the SISMER clinic | Men with OAT with deficiencies in all sperm patterns (sperm conc <20,000000/mL, class A motility, typical forms <30%), with main problem of primary infertility longer than 12 months, regular intercourse. normal sperm appearance, consistency, liquefaction, volume, pH, female partner with no fertility problems (confirmed with history, examination, body temp recording, progesterone levels, ultrasound and hysterosalpingogram). Exclusion: azoospermia, seminal WBC conc >1000000/mL, positive seminal cultural analysis, positive urethral swab chlamydia test, oligospermia less than 5,000000mL, hormonal alterations, age more than 40 years, presence of anti-sperm antibodies, drug, tobacco or alcohol abuse, ongoing medical treatments eg. gonadotropins, cancer, NSAIDs, presence of hydrocele, diabetes, hypertension, x-ray exposure in prev 8 months, peptic ulcer, unexplained gastric pain, previous hypersensitivity to NSAIDs or carnitines, carnitine metabolism deficiency, bilateral variocele, prostate abnormalities (see criteria), previous or concurrent testicular pathology e.g. torsion, trauma or testicle echographic abnormalities. |
| Cochrane 2016 | 23.2% | Contacts within research center, newspaper ads, Facebook ads, forums on fertility specific websites, posters and pamphlets in university campuses, medical centers, chemists and community centers, letter requesting referrals to women's health centers, medical centers, TCM clinics, fertility centers | women age 18-44 who had been trying unsuccessfully to conceive for 12 months (inc. miscarriage), had gynaecological diagnosis for cause of infertility, not planning to use acupuncture during the trial, able to attend 7/9 sessions. excluded: nonpatent fallopian tubes, absence of uterus, primary anovulation, partner sperm defect |
| Gopinath 2013 | 9.4% | Selected at fertility clinics of five centres across India | Male patients aged 21-50 presenting with primary infertility >1year, sperm count <15million/ml, sperm total motility <40%, no history of taking therapy for infertility, no history of obstructive azoospermia, regular sexual intercourse with a potentially normal fertile female. exclude: primary testicular disease, organic cause for infertility inc. varicocele, prostato-vesiculo-epidiymitis, genital infectious disease, planning ofr any other ART during study period, serum follicle stimulating hormone FSH >15mIU/ml, abnormal serum levels of LH, testosterone, estradiol and prolactin, presence of antispermatozoa antibodies, severe olgiospermia (<2million sperm/ml), azoospermia, seminal WBCs more than 1*10^6, major hepatic and renal disease, myopathy, history of allergy to any ingredient of the formulation, not likely to be available for follow-up, participated in another clinical trial in last 3 months, female partners with anatomic or physiological alterations causing subfertility |
| Holt 2009 | 18.4% | Recruited from South West Centre for Reproductive Medicine at Derriford Hospital, Plymouth, later Exeter and Torbay recruited | females 18-38y with anovulation (<6 menstrual periods in last 12 months or luteal phase progesterone <28nmol/L). biochemical evidence of raised androgens inkeeping with diagnostic criteria for PCOS. exclusion: diabetes mellitus, thyroid disease, late onset adrenal hyperplasia, use of ovulation inducing agents with past 2 months, previous reflexology treatment, pregnancy, active thrombosis or phlebitis, foot deformity, recent sprain or trauma to ankles or feet, infections or inflammatory conditions of the feet, topical steroid use of longer than 6 months, pacemaker. BMI> or equal to 35. |
| Lenzi 2003 | 14% | Recruited from a pool of 1000 patients presenting to clinic | Inc: Males aged 20-40 , infertility longer than 2 years, regular sexual intercourse, gynaecologically normal partner (assess using temp, luteal progesterone, ultrasound and histerosalpingogram). Absence of general and endocrinological diseases, no present or previous cryptochidism, no genital infections or genital tract obstructions, no varicocele and testicular hypertrophy, no antisperm antibodies. seminological inclusion criteria: normal rheological characteristics, normal volume and pH. sperm conc 10-20*10^6mL, total motility 10%-30%, forward motility <15%, atypical forms <70%,semen leukocytes <1*10^6mL, sperm velocity 10-30u/s and linearity <4. overall for inclusion patients had to meet the seminological inclusion criteria at time of first control and maintain this for further two washout controls as well as show no statistically significant differences in the 3 evaluations before treatment. |
| Lenzi 2004 | 6.70% | Selected by an andrology team | Male, age 20-40, infertility >2y, regular sexual intercourse with a gynecologically normal partner (verified using biphasic basal body temperature, P levels in luteal phase, U/S ovary and uterus evaluation, and hysterosalpingogram to assess tubal patency). Seminological criteria must have normal rheology, volume, pH, sperm concentration 10-20x10^6/mL, motility 10-30%, forward motility <15%, atypical forms <80%, semen leukocytes <1x10^6/mL. These criteria had to be met at the T-2m screening visit and T+0 |
| Lim 2014 | 2.70% | Unknown | Women with PCOS (Rotterdam criteria) and kidney yang deficiency +/- phlegm +/- blood stasis |
| Pastore 2011 | 11.6% | Women living in the state of Virginia were recruited using posters, flyers and direct mailing | PCOS and no hormonal intervention 60d before enrollment |
| Razavi 2016 | 6.25% | Women presenting to clinics at the Ardabil University of Medical Sciences | Women aged 18-40 with a diagnosis of PCOS (defined by oligomenorrhoea + high androgens). >1 menses in past 6 months, <8 menses in last 12 months without hormonal intervention. Agreement to not take hormonal contraceptives, metformin or fertility medication for 5 months during the study. |
| Scott 1998 | 7.2% | Selected from men attending the Combined Subfertility Clinic in Glasgow Royal Infirmary | Semen samples showing reduces sperm motility. |
| Sigman 2006 | 19.2% | Unknown | Males aged 18 - 65 years with infertility of > 6 months, sperm concentration of > 5x10^6 sperm/mL, motility of 10%–50%, no pyospermia, and normal FSH and testosterone levels |
| Westphal 2004 | 0% | Unknown | Women aged 24-46 years who had tried to conceive unsuccessfully for 6-36 months |
| Wittman 1993 | 3.6% | Unknown | Women over 18 who wish to have children, tubal patency, positive post coital test, normal spermiogram |
| Wu 2017 | 7.4% | Multicenter study, recruited via posters/flyers, direct mailings, recruitment by investigators from their practices/continuity clinics | Age of woman 20-40 y; confirmed PCOS according to modified Rotterdam criteria + anovulation + PCOS or hyperandrogenism; patency of > 1 tube and a normal uterine cavity; semen analysis showing a sperm concentration >15x10^6/ml and total motility >40% or forward motility >32% and total motile sperm count > 9 million |
| Zavacki 2003 | 23.0% | Presented at the Andrology Division of Department of Obstetrics and Gynaecology, University of Szeged for infertility problems | Men who have been attempting to conceive for >1 year; healthy female partners examined by gynaecologists; diagnosis of pathospermia according to WHO 1992 laboratory manual (in two consecutive ejaculations, ejaculate volume < 2 ml and/or sperm concentration<20 million/ml and/or motility < 50% and/or normal morphology ratio <30%); no detectable organic alteration of reproductive organs from physical and ultrasound examinations; no signs or symptoms of genital tract infections; all spermatogenesis related hormones (FSH, LH, prolactin, testosterone) are within physiological range; intact renal function (blood serum creatinine level <120 µmol/l); no excess magnesium intake 4 weeks prior to initiation of study |

## eTable 5: Results of studies evaluating conception in relation to a complementary or alternative medicine.

| Study, year | Complementary or alternative medicine | Control group intervention | Control group (N) | | Therapy group 1 (N) | Therapy group 2 (N) | Therapy group 3 (N) | Pregnancy, N (%) | | | | Risk estimate |
| --- | --- | --- | --- | --- | --- | --- | --- | --- | --- | --- | --- | --- |
|  |  |  |  |  |  |  |  | Control | Therapy group 1 | Therapy group 2 | Therapy group 3 |  |
| Arentz 2017 | Tablet with Glycyrrhiza glabra, Peonia lactiflora, Cinnamonum verum and Hypericum perforatum plus a tablet with Tribulus terrestris extract during the follicular phase of the menstrual cycle, & lifestyle intervention. | Lifestyle intervention only. | 36 | | 34 | - | - | 8.3% | 32.4% | - | - | p=0.012 |
| Balercia 2005 | L-carnitine (syrup), L-acetylcarnitine (tablets) or combined L-carnitine and L-acetylcarnitine. Group 1 - LC syrup 10mL phial of 3g/d oral. GRoup 2 - LAC 3g/d oral tablets. Group 3 - both LC syrup (2g/d oral) and LAC tablets (1g/d oral). Tablet = Zibren (name) | 2 x 10ml placebo vials & 1 x placebo tablet od. Placebo vial contained malic acid, sodium benzoate, sodium saccharinate dihydrate, anhydrous sodium citrate, pineapple flavouring, & demineralized water. Placebo tablet contained 1-hydro lactose, magnesium stearate, corn starch polyvinylpyrrolidone, & coating of cellulose acetophtalate, dimethicone & ethylphtalate. | 15 | | 15 | 15 | 15 | 20.0% | 13.3% | 13.3% | 33.3% | p=0.624 / p=0.624 / p=0.409 |
| Balercia 2009 | 100mg of CoQ10, lecithin and medium chain glycerides. | Soft gels with lecithin, medium chain glycerides (no CoQ10) | 30 | | 30 | - | - | 10.0% | 20.0% | - | - | p=0.278 |
| Bergmann 2004 | Phyto Hypophyson drops - an Agnus castus-containing homeopathic preparation. Tincture containing vitex agnus caius, chelidonium majus, & silybum marianum. 50 drops taken tds for 3 months or 3 menstrual cycles. | 50 drops/day of placebo tds over 3 months/menstrual cycles. Placebo was 40% w/v alcohol solution in water | 30 | | 30 | - | - | 3.3% | 6.7% | - | - | p=0.554 |
| Busetto 2018 | Supplement containing 1000mg L-carnitine, 725mg fumarate, 500mg acetyl-l-carnitine, 1000mg fructose, 20mg CoQ10, 90mg vitamin C, 10mg zinc, 200ug folic acid and 1.5ug vitamin B12. + excipients (sucrose, silica (anti-caking), lemon flavour, acesulfame K (E950) sweetener). 2 sachets daily for 6 months. | Placebo from same company. just made with the excipients - sucrose, silica (anti-caking), lemon flavour, acesulfame K (E950) sweetener. two sachets daily for 6 months | 45 | | 49 | - | - | 4.4% | 20.4% | - | - | p=0.021 |
| Cavallini 2004 | Group 1 - oral L-carnitine (1x2g/d) + oral acetyl-l-carnitine (2x500mg/d) + 1 glycerine suppository every 4 days.  Group 2 - L-carnitine/acetyl-l-carnitine + 1x30mg cinnoxicam suppository every 4 days. | starch 500mg tablets and commercial glycerine suppositories (glicerolo supposte 2500mg). placebo group (G1) had 1 starch tablet twice daily and 1 glycerine suppository every 4 days | 118 | | 101 | 106 | - | 1.7% | 21.8% | 38.0% | - | p<0.001 / p<0.001 |
| Cochrane 2016 | 3 months of weekly acupuncture, & lifestyle advice, including diet, exercise, & removal of cigarettes, alcohol or regular caffeine. Acupuncture was tailored to stage of menstrual cycle, diagnosis from traditional Chinese medicine view, assessment of emotional state, biomedical condition. Needled bilaterally on body channels apart from unilateral on channels that bisect the midline. Needles in for 20-30 mins each session. | 3 months lifestyle intervention. diet based on CSIRO total wellbeing diet. exercise program within guidelines. remove cigarettes, alcohol or regular caffeine. | 28 | | 28 | - | - | 17.9% | 35.7% | - | - | p=0.131 |
| Gopinath 2013 | Group 1 - 2 tablets of antioxidants twice daily. The tablet contained (Co-Q10 50mg, L-carnitine 500mg, zinc 12.5mg and lycopene 2.5mg). Group 2 - 1 tablet + 1 placebo tablet twice daily. All for 180 days | 2 tablets of placebo twice daily. for 180 days | 36 | | 46 | 43 | - | 5.6% | 13.0% | 16.3% | - | p=0.257 / p=0.135 |
| Holt 2009 | Reflexology (Bayly method) seven sessions of 1h intervention over 6 weeks, then one treatment one month later | Gentle foot massage avoiding specific areas. seven sessions over 6 weeks, one treatment one month later | 18 | | 21 | - | - | 11.1% | 19.0% | - | - | p=0.494 |
| Lenzi 2003 | 2 months of 2g/d orally of l-carnitine. study design - 2 months washout, 2 months therapy, 2 months washout, 2 months therapy, 2 months follow up. | equal volume of identical placebo | - * | 86 | | - | - | 0.0% | 9.3% | - | - | p=0.004 |
| Lenzi 2004 | 6 months of L-carnitine (10 mL vial containing Carnitene) to be taken 2g/d orally and L-acetyl-carnitine (tablet containing 500 mg), to be taken orally bd | 10-mL placebo vial contains malic acid, sodium benzoate, sodium saccharinate dihydrate, anhydrous sodium citrate, pineapple flavoring, demineralized water) to be taken 2g/d for 6 months, each placebo tablet contains a core with 1-hydro lactose, magnesium stearate, polyvinylpyrrolidone, corn starch, and a coating with cellulose acetophthalate, dimethicone, ethyl phthalate to be taken orally bd | - * | | 60 | - | - | 0.0% | 13.3% | - | - | p=0.045 |
| Lim 2014 | 3 months of acupuncture. Weekly session for 20 minutes using 0.26mm needles. On achieving the arrival of Qi, the needles were toned by rotating the needles evenly in a clockwise direction, and the body of each needle was scraped with a fingernail every 10 minutes. Blinding was achieved by placing a curtain between the acupuncturist and the patient. | Weekly sham acupuncture for 3 months. Acupuncture was done at points around 0.5-1cm outside of the classical points and outside of the meridian. No stimulation was used. | 48 | | 98 | - | - | 0.0% | 5.1% | - | - | p=0.002 |
| Pastore 2011 | 12 sessions of acupuncture over 8 weeks | 12 sessions of sham acupuncture (Park sham device, avoiding standard meridians and points) over 8 weeks | 44 | | 40 | - | - | 12.0% | 20.0% | - | - | p=0.680 |
| Razavi 2016 | 200 ug selenium od for 8 weeks + 500 mg metformin od, & increased by 500mg per week for 3 weeks, then maintained until week 8. | Placebo od for 8 weeks + 500 mg metformin od, & increased by 500mg per week for 3 weeks, then maintained until week 8. | 32 | | 32 | - | - | 3.1% | 18.8% | - | - | p=0.040 |
| Scott 1998 | Group 1: 100ug/d of selenium as L-selenomethionine at night for 3 months  Group 2: 100 ug/d L-selenomethionine + 1 mg vitamin A + 10 mg vitamin C + 15 mg vitamin E at night for 3 months | 1 tab nocte for 3 mths consisting of placebo | 18 | | 16 | 30 | - | 0.0% | 10.9% (breakdown unavailable) | | - | p=0.145 |
| Sigman 2006 | 1000 mg L-carnitine + 500 mg L-acetyl-carnitine taken orally BD for four months | Placebo taken orally BD for four months | 9 | | 12 | - | - | 11.1% | 0.00% | - | - | p=0.237 |
| Westphal 2004 | A proprietary nutritional supplement containing chasteberry and green tea extracts, L-arginine, vitamins (E, B6, B12 and folate) and minerals (iron, magnesium, zinc, selenium). 3 capsules/day. | 3 months of placebo, then 3 months of the nutritional supplement. | 15 | | 15 | - | - | 0.0% | 33.3% | - | - | p=0.014 |
| Wittman 1993 | 2x 30 drops Mastodynon daily over 3 months | 2x 30 drops placebo daily over 3 months | 42 | | 39 | - | - | 21.4% | 23.1% | - | - | p=0.494 |
| Wu 2017 | Manual & electrical stimulation acupuncture for 30mins 2x weekly for up to 16 weeks.  1 x 50mg od clomiphene during days 3-7 of menstrual cycle, and dosage was increased by 1 pill if no ovulation occurred to a maximum of 150mg/day or 750mg/cycle, for up to 4 cycles.  Group 1: Active acupuncture + clomiphene Group 2: Control acupuncture + clomiphene Group 3: Active acupuncture + placebo | Control acupuncture + placebo. Control acupuncture needles were inserted in non-acupuncture points and no stimulation given. | 232 | | 235 | 236 | 223 | 17.7% | 29.4% | 28.0% | 13.9% | Risk difference (95%CI) for the effect of active acupuncture: plus clomphene: 1.8 (-6.5 to 10.1); plus placebo: -2.9 (-9.6 to 3.9); overall: -0.4 (-5.8 to 5.1) |
| Zavacki 2003 | Six tablets (3 × 2) of Magnesium-orotate daily for 90d for a total daily magnesium-orotate intake of 3g (equivalent to 196.8 mg pure magnesium). | Placebo for 90d | 10 | | 10 | - | - | 0.0% | 10.0% | - | - | p=0.305 |

** Cross over trial in which participants had period of time with and without treatment*

*bd – twice daily, d – daily, g – grams, mg – milligrams, nocte – at night, od – once daily, tds – three times a day, ug = micrograms*

## eTable 6: Results of studies evaluating miscarriages in relation to a complementary or alternative medicine.

| Study, year | Complementary or alternative medicine | Control group intervention | Control group (N) | Therapy group 1 (N) | Therapy group 2 (N) | Therapy group 3 (N) | Miscarriages, N (%) | | | | Risk estimate |
| --- | --- | --- | --- | --- | --- | --- | --- | --- | --- | --- | --- |
|  |  |  |  |  |  |  | Control | Therapy group 1 | Therapy group 2 | Therapy group 3 |  |
| Arentz 2017 | Tablet with Glycyrrhiza glabra, Peonia lactiflora, Cinnamonum verum and Hypericum perforatum plus a tablet with Tribulus terrestris extract during the follicular phase of the menstrual cycle, & lifestyle intervention. | Lifestyle intervention only | 36 | 34 | - | - | 1/3 (33%) | 4/11 (36%) | - | - | p=0.923 |
| Westphal 2004 | A proprietary nutritional supplement containing chasteberry and green tea extracts, L-arginine, vitamins (E, B6, B12 and folate) and minerals (iron, magnesium, zinc, selenium). 3 capsules/day. | 3 months of placebo, then 3 months of the nutritional supplement. | 15 | 15 | - | - | n/a * | 1/5 (20%) | - | - | - |
| Wu 2017 | Manual & electrical stimulation acupuncture for 30mins 2 x weekly for up to 16 weeks.  1 x 50mg od clomiphene during days 3-7 of menstrual cycle, and dosage was increased by 1 pill if no ovulation occurred to a maximum of 150mg/day or 750mg/cycle, for up to 4 cycles.  Group 1: Active acupuncture + clomiphene Group 2: Control acupuncture + clomiphene Group 3: Active acupuncture + placebo | Control acupuncture + placebo. Control acupuncture needles were inserted in non-acupuncture points and no stimulation given. | 232 | 235 | 235 | 223 | 16/55 (29.1%) | 38/108 (35.2%) | 37/106 (34.9%) | 19/51 (37.3%) | Risk difference (95%CI) for the effect of active acupuncture: plus clomphene: 0.3 (-12.5 to 13.1);plus placebo: 8.2 (-9.7 to 26.1); overall: 2.9 (-7.5 to 13.3) |

** not applicable as no pregnancies*

*bd – twice daily, d – daily, g – grams, mg – milligrams, nocte – at night, od – once daily, tds – three times a day, ug = micrograms*

## eTable 7: Results of studies evaluating live-births in relation to a complementary or alternative medicine.

| Study, year | Complementary or alternative medicine | Control group intervention | Control group (N) | Therapy group 1 (N) | Therapy group 2 (N) | Therapy group 3 (N) | Live births, N (%) | | | | Risk estimate |
| --- | --- | --- | --- | --- | --- | --- | --- | --- | --- | --- | --- |
|  |  |  |  |  |  |  | Control | Therapy group 1 | Therapy group 2 | Therapy group 3 |  |
| Arentz 2017 | Tablet with Glycyrrhiza glabra, Peonia lactiflora, Cinnamonum verum and Hypericum perforatum plus a tablet with Tribulus terrestris extract during the follicular phase of the menstrual cycle, & lifestyle intervention. | Lifestyle intervention only. | 36 | 34 | - | - | 5.6% | 20.6% | - | - | p=0.060 |
| Bergmann 2004 | Phyto Hypophyson drops - an Agnus castus-containing homeopathic preparation. Tincture containing vitex agnus caius, chelidonium majus, & silybum marianum. 50 drops taken tds for 3 months or 3 menstrual cycles. | 50 drops/day of placebo tds over 3 months or menstrual cycles. Placebo was 40% w/v alcohol solution in water | 30 | 30 | - | - | 6.67% | 20.00% | - | - | p=0.129 |
| Westphal 2004 | A proprietary nutritional supplement containing chasteberry and green tea extracts, L-arginine, vitamins (E, B6, B12 and folate) and minerals (iron, magnesium, zinc, selenium). 3 capsules/day. | 3 months of placebo, then 3 months of the nutritional supplement. | 15 | 15 | - | - | 0.00% | 26.67% | - | - | p=0.032 |
| Wu 2017 | Manual & electrical stimulation acupuncture for 30mins 2x weekly for up to 16 weeks.  1 x 50mg od clomiphene during days 3-7 of menstrual cycle, and dosage was increased by 1 pill if no ovulation occurred to a maximum of 150mg/day or 750mg/cycle, for up to 4 cycles.  Group 1: Active acupuncture + clomiphene Group 2: Control acupuncture + clomiphene Group 3: Active acupuncture + placebo | Control acupuncture + placebo. Control acupuncture needles were inserted in non-acupuncture points and no stimulation given. | 232 | 235 | 236 | 223 | 16.81% | 29.36% | 27.97% | 13.90% | Risk difference (95%CI) for the effect of active acupuncture: plus clomphene: 1.4 (-6.8 to 9.6); plus placebo: -2.9 (-9.5 to 3.7); overall: -0.6 (-5.9 to 4.7) [Overall: 100 of 458 [21.8%] vs 105 of 468 [22.4%]] |

*bd – twice daily, d – daily, g – grams, mg – milligrams, nocte – at night, od – once daily, tds – three times a day, ug = micrograms*

## eTable 8: Quality of studies in the review as assessed by the Cochrane Collaboration’s tool.

| Paper | Random sequence generation | Allocation concealment | Selective reporting | Blinding participants & personnel | Blinding outcome assessment | Incomplete outcome data | Other sources of bias | Overall |
| --- | --- | --- | --- | --- | --- | --- | --- | --- |
| Arentz (2017) | Low | Low | Unclear | High | Low | Low | Low | High |
| Balercia (2005) | Unclear | Unclear | Unclear | Unclear | Unclear | Unclear | Low | Unclear |
| Balercia (2009) | Unclear | Unclear | Unclear | Unclear | Unclear | Unclear | Low | Unclear |
| Bergmann (2004) | Unclear | Unclear | Unclear | Unclear | Low | Low | Low | Unclear |
| Busetto (2018) | Low | Unclear | Unclear | Unclear | Unclear | Unclear | Low | Unclear |
| Cavallini (2004) | Unclear | Unclear | Unclear | Low | Low | Low | Low | Unclear |
| Cochrane (2016) | Low | Low | Unclear | High | Low | Low | Low | High |
| Gopinath (2013) | Unclear | Unclear | Unclear | Unclear | Unclear | Unclear | Low | Unclear |
| Holt (2009) | Low | Low | Low | Low | Low | Low | Low | Low |
| Lenzi (2003) | Unclear | Unclear | Unclear | Unclear | Unclear | Low | Low | Unclear |
| Lenzi (2004) | Unclear | Unclear | Unclear | Unclear | Unclear | Unclear | Low | Unclear |
| Lim (2014) | Low | Low | Unclear | Unclear | Unclear | Low | Low | Low |
| Pastore (2011) | Low | Low | Low | Low | Low | Low | Low | Low |
| Rasavi (2015) | Low | Low | Unclear | Low | Low | Low | Low | Low |
| Scott (1998) | Low | Unclear | Unclear | Unclear | Unclear | Low | Low | Unclear |
| Sigman (2006) | Unclear | Unclear | Unclear | Unclear | Unclear | Low | Low | Unclear |
| Westphal (2004) | Unclear | Unclear | Unclear | Unclear | Unclear | Low | Low | Unclear |
| Witmann (1993) | Unclear | Unclear | Unclear | Unclear | Low | Unclear | Unclear | Unclear |
| Wu (2017) | Low | Low | Unclear | Low | Low | Low | Low | Low |
| Zavacki (2003) | Unclear | Unclear | Unclear | Unclear | Unclear | Low | Low | Unclear |
